# Highly accurate blood test for Alzheimer’s disease is similar or superior to clinical cerebrospinal fluid tests

**DOI:** 10.1038/s41591-024-02869-z

**Published:** 2024-02-21

**Authors:** Nicolas R. Barthélemy, Gemma Salvadó, Suzanne E. Schindler, Yingxin He, Shorena Janelidze, Lyduine E. Collij, Benjamin Saef, Rachel L. Henson, Charles D. Chen, Brian A. Gordon, Yan Li, Renaud La Joie, Tammie L. S. Benzinger, John C. Morris, Niklas Mattsson-Carlgren, Sebastian Palmqvist, Rik Ossenkoppele, Gil D. Rabinovici, Erik Stomrud, Randall J. Bateman, Oskar Hansson

**Affiliations:** 1 Department of Neurology, Washington University School of Medicine, St. Louis, MO USA; 2 Tracy Family Stable Isotope Labeling Quantitation (SILQ) Center, Washington University School of Medicine, St. Louis, MO USA; 3 Clinical Memory Research Unit, Department of Clinical Sciences Malmö, Faculty of Medicine, Lund University, Lund, Sweden; 4 Charles F. and Joanne Knight Alzheimer Disease Research Center (Knight ADRC), Washington University School of Medicine, St. Louis, MO USA; 5 Department of Radiology and Nuclear Medicine, Amsterdam UMC, location VUmc, Amsterdam, The Netherlands; 6 Amsterdam Neuroscience, Brain Imaging, Amsterdam, The Netherlands; 7 Department of Radiology, Washington University School of Medicine, St. Louis, MO USA; 8 Division of Biostatistics, Washington University in St. Louis, St. Louis, MO USA; 9 Department of Neurology, Memory and Aging Center, Weill Institute for Neurosciences, University of California, San Francisco, San Francisco, CA USA; 10 Wallenberg Center for Molecular Medicine, Lund University, Lund, Sweden; 11 Department of Neurology, Skåne University Hospital, Lund University, Lund, Sweden; 12 Memory Clinic, Skåne University Hospital, Malmö, Sweden; 13 Alzheimer Center Amsterdam, Neurology, Vrije Universiteit Amsterdam, Amsterdam UMC, location VUmc, Amsterdam, The Netherlands; 14 Department of Radiology and Biomedical Imaging, University of California, San Francisco, San Francisco, CA USA

**Keywords:** Diagnostic markers, Alzheimer's disease, Diagnosis

## Abstract

With the emergence of Alzheimer’s disease (AD) disease-modifying therapies, identifying patients who could benefit from these treatments becomes critical. In this study, we evaluated whether a precise blood test could perform as well as established cerebrospinal fluid (CSF) tests in detecting amyloid-β (Aβ) plaques and tau tangles. Plasma %p-tau217 (ratio of phosporylated-tau217 to non-phosphorylated tau) was analyzed by mass spectrometry in the Swedish BioFINDER-2 cohort (*n* = 1,422) and the US Charles F. and Joanne Knight Alzheimer Disease Research Center (Knight ADRC) cohort (*n* = 337). Matched CSF samples were analyzed with clinically used and FDA-approved automated immunoassays for Aβ42/40 and p-tau181/Aβ42. The primary and secondary outcomes were detection of brain Aβ or tau pathology, respectively, using positron emission tomography (PET) imaging as the reference standard. Main analyses were focused on individuals with cognitive impairment (mild cognitive impairment and mild dementia), which is the target population for available disease-modifying treatments. Plasma %p-tau217 was clinically equivalent to FDA-approved CSF tests in classifying Aβ PET status, with an area under the curve (AUC) for both between 0.95 and 0.97. Plasma %p-tau217 was generally superior to CSF tests in classification of tau-PET with AUCs of 0.95–0.98. In cognitively impaired subcohorts (BioFINDER-2: *n* = 720; Knight ADRC: *n* = 50), plasma %p-tau217 had an accuracy, a positive predictive value and a negative predictive value of 89–90% for Aβ PET and 87–88% for tau PET status, which was clinically equivalent to CSF tests, further improving to 95% using a two-cutoffs approach. Blood plasma %p-tau217 demonstrated performance that was clinically equivalent or superior to clinically used FDA-approved CSF tests in the detection of AD pathology. Use of high-performance blood tests in clinical practice can improve access to accurate AD diagnosis and AD-specific treatments.

## Abstract (web summary)

The performance of plasma %p-tau217 is clinically equivalent in classification of Aβ PET status and superior in classification of tau PET status compared to clinically used and FDA-approved CSF tests in cognitively impaired participants.

## Main

Dementia affects more than 40 million people worldwide^1^, and its prevalence is projected to rise to 130 million by the year 2050^2^. The annual global cost associated with dementia is approximately $1 trillion US dollars^2^, making it a major global contributor to disability, institutionalization and mortality. Alzheimer’s disease (AD) accounts for 60–70% of all dementia cases^3^ and is characterized by the deposition of amyloid-β (Aβ)-containing plaques in the extracellular space of the brain parenchyma and the formation of intraneuronal tau tangle aggregates. During an extended pre-symptomatic phase, which lasts 10–20 years, Aβ plaques first accumulate in the cortex and are thought to facilitate the subsequent spread of tau pathology from the medial temporal lobe to neocortical areas^4^. The presence of tau pathology in the neocortex is correlated with the clinical phase of the disease, which is marked by progressive cognitive impairment and dementia^5^.

Several phase 3 trials demonstrated that anti-amyloid antibodies can clear Aβ plaques from the brain^6–8^, which leads to a slowing of cognitive and functional decline in individuals with mild cognitive impairment (MCI) and mild dementia due to AD. Recently, lecanemab received traditional approval from the US Food & Drug Administration (FDA) for treatment of patients with MCI and mild dementia with biomarker-proven Aβ pathology^8^, and other immunotherapies are expected to follow. The presence of Aβ pathology can be determined by positron emission tomography (PET), which visualizes Aβ deposition in the brain, or cerebrospinal fluid (CSF) assays, which measure CSF levels of Aβ42 as a ratio with Aβ40, phosphorylated tau (p-tau) or total tau^4,9–11^. Biomarker testing reduces dementia misdiagnoses: when biomarkers are not used, the rate of misdiagnosis is approximately 25–35% in specialty clinics and even higher in primary care clinics^4,12,13^. Additionally, PET and CSF can identify cognitively unimpaired individuals at high risk of future cognitive decline and progression to AD dementia^14,15^. However, although safe, the widespread clinical use of PET and CSF has been hampered by high costs, reliance on expensive equipment and specially trained personnel and perceived invasiveness^11^. As a result, there is an urgent need for scalable and cost-effective methods to detect AD pathology in routine clinical practice.

In the last several years, blood-based markers (BBMs) capable of detecting AD pathology have been developed^16–18^. Plasma levels of p-tau are strongly associated with PET and CSF biomarkers of AD pathology^19–25^, neuropathological changes associated with AD^20,23,26,27^ and the subsequent development of AD dementia^20,23,28^. Among different p-tau variants, tau phosphorylated at threonine 217 (p-tau217) has demonstrated the highest accuracy in detecting AD pathology and predicting future cognitive decline^23,27,29–31^. However, certain comorbidities, especially kidney disease, can lead to false elevations in plasma p-tau levels^32,33^, although this can be mitigated by using the ratio of p-tau217 to the non-phosphorylated levels of the same tau peptide (%p-tau217)^34^. Potentially because %p-tau217 is less affected by confounding factors, this blood test has the highest performance yet demonstrated in identifying individuals with AD pathology^29^.

Despite BBMs being used in clinical practice in some countries, including the United States, they have not been recommended as standalone diagnostic tests due to a lack of studies demonstrating their equivalence to clinically used CSF and PET methods^16,35–37^. Therefore, we compared the diagnostic performance of plasma %p-tau217 with clinically used and FDA-approved CSF assays (CSF Aβ42/40 from Fujirebio and p-tau181/Aβ42 from Roche) in independent Swedish and US cohorts. Because confirmation of Aβ positivity is required for initiation of anti-amyloid immunotherapies, the primary outcome was the detection of Aβ pathology as determined by Aβ PET imaging. Secondary outcomes included the classification of brain tau aggregates as determined by tau PET imaging, which has also been used by some trials in the selection of patients suitable for anti-amyloid immunotherapy^7,38^, and agreement with a clinical AD diagnosis. Our main analyses were focused on individuals with cognitive impairment (MCI and mild dementia), because the clinical use of anti-amyloid therapies is currently approved for cases where cognitive impairment is deemed to be caused by AD pathology.

## Results

### Study participants

The BioFINDER-2 cohort included 1,422 participants with a mean (standard deviation (s.d.)) age of 69.3 (10.6) years, of whom 708 (49.8%) were female and 702 (49.3%) were cognitively impaired as defined by either MCI or dementia (Table 1). The Charles F. and Joanne Knight Alzheimer Disease Research Center (Knight ADRC) cohort included 337 participants with a mean age of 69.8 (8.3) years, of whom 175 (51.9%) were female and 50 (14.8%) were cognitively impaired.

**Table 1:** Participant characteristics

	BioFINDER-2			Knight ADRC		
	All (*n* = 1,422)	Cognitively unimpaired (*n* = 720)	Cognitively impaired (*n* = 702)	All (*n* = 337)	Cognitively unimpaired (*n* = 287)	Cognitively impaired (*n* = 50)
Age, years	69.3 (10.6)	66.3 (12.1)	72.3 (7.9)	69.8 (8.3)	69.0 (8.3)	74.6 (6.5)
Women, *n* (%)	708 (49.8%)	393 (54.6%)	315 (44.9%)	175 (51.9%)	155 (54.0%)	20 (40.0%)
*APOE*-ε4 carriers, *n* (%)^a^	659 (51.5%)	278 (47.8%)	381 (54.6%)	128 (38.0%)	101 (35.2%)	27 (54.0%)
Years of education^b^	12.7 (3.9)	12.9 (3.6)	12.4 (4.2)	16.4 (2.4)	16.5 (2.3)	15.6 (2.6)
Race (Black/White/Other), *n*	N.A.	N.A.	N.A.	24/308/5	24/259/4	0/49/1
MMSE^c^	26.7 (3.9)	28.9 (1.2)	24.4 (4.4)	28.8 (2.1)	29.3 (1.1)	26.1 (3.7)
Aβ PET, Centiloids^d^	19.2 (41.8)	7.99 (31.9)	45.0 (49.7)	25.0 (35.6)	17.3 (27.2)	69.3 (44.8)
Aβ PET positive, *n* (%)^d^	258 (25.8%)	107 (15.3%)	151 (49.7%)	85 (25.2%)	48 (16.7%)	37 (74.0%)
Tau PET, SUVR^e^	1.36 (0.47)	1.18 (0.17)	1.56 (0.59)	1.20 (0.19)	1.16 (0.09)	1.47 (0.35)
Tau PET positive^e^	355 (25.0%)	49 (6.8%)	306 (43.6%)	35 (10.4%)	7 (2.4%)	28 (56.0%)
AD diagnosis, *n* (%)	346 (24.3%)	0 (0%)	346 (49.3%)	50 (14.8%)	0 (0%)	50 (100%)
Severity of cognitive impairment (CU/MCI/dementia)	720/366/336	720/0/0	0/366/336	287/37/13	287/0/0	0/37/13

All measures represent mean (s.d.) unless otherwise stated. Percentages are calculated from the sample available for each variable. Aβ PET positivity was defined as Centiloids ≥ 37. Tau PET positivity was defined using previously validated in-house thresholds (SUVR > 1.32 for both cohorts). Race was not collected in the BioFINDER-2 cohort.

^a^: 143 participants missing in BioFINDER-2

^b^: 31 participants missing in BioFINDER-2

^c^: 1 participant missing in BioFINDER-2

^d^: 421 participants missing in BioFINDER-2

^e^: 54 participants missing in BioFINDER-2

^f^: 432 participants missing in BioFINDER-2

CU, cognitively unimpaired; MCI, mild cognitive impairment; MMSE, Mini-Mental State Examination; N.A., not applicable; SUVR, standardized uptake value ratio.

### Classification of Aβ or tau PET status by fluid biomarkers

**Fig. 1:**
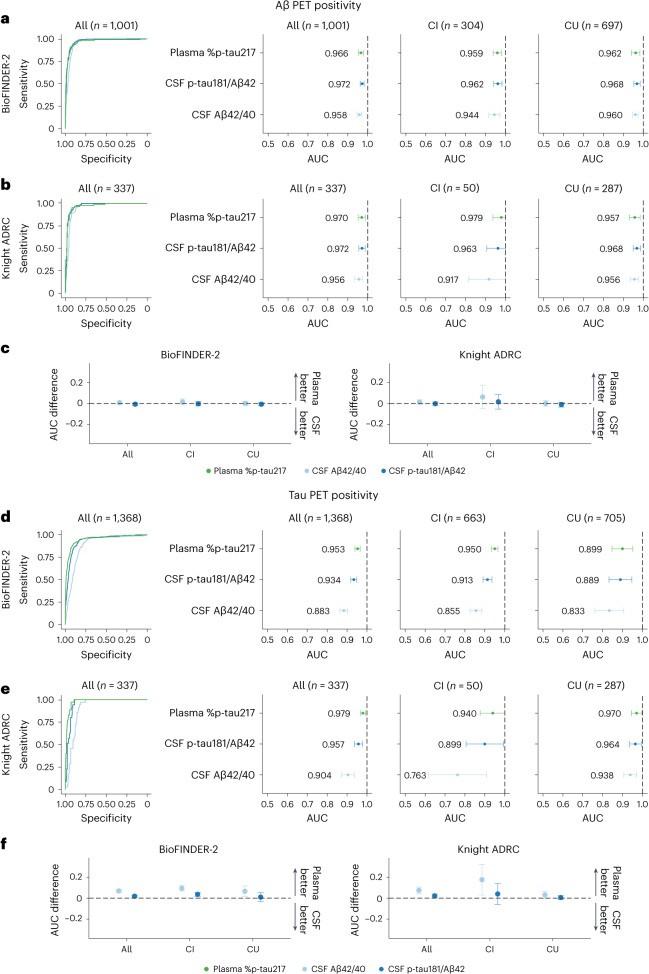
Concordance of fluid and imaging biomarkers of amyloid and tau pathologies. **a**,**b**,**d**,**e**, Concordance of fluid biomarkers with Aβ and tau PET positivity in BioFINDER-2 (**a** and **d**) and Knight ADRC (**b** and **e**) participants. ROC curves including all participants are included in the first row. AUCs for all, cognitively impaired and cognitively unimpaired groups are shown in the next three columns, respectively. **c**,**f**, Bootstrapped differences (*n* = 1,000 resamples with replacement stratifying by the output) between the statistics using plasma %p-tau217 (reference) and CSF biomarkers are shown in **c** and **f** for both the BioFINDER-2 cohort (left) and the Knight ADRC (right) cohort. The horizontal dashed line is plotted at zero, representing the lack of difference between plasma and CSF biomarkers. We considered plasma and CSF biomarkers clinically equivalent if the 95% CI of the mean difference included zero and clinically superior if it did not include zero and favored plasma (>0). Dots and error bars represent the actual statistic and 95% CI (from bootstrapped *n* = 1,000 samples with replacement), respectively. Vertical dashed lines represent the maximal AUC value possible (1). Aβ PET positivity was assessed as Centiloids ≥ 37. Tau PET positivity was assessed using previously validated in-house thresholds (SUVR > 1.32 in Braak I–IV for both cohorts). AUC, area under the curve; CI, cognitively impaired; CSF, cerebrospinal fluid; CU, cognitively unimpaired; SUVR, standardized uptake value ratio; CI, confidence interval.

We first compared the area under the curve (AUC) of plasma %p-tau217 with clinically used CSF biomarkers in classification of Aβ PET (Centiloids ≥ 37) or tau PET status (standardized uptake value ratio (SUVR) > 1.32 in Braak I–IV region of interest (ROI) for both cohorts) (Fig. 1 and Extended Data Table 1). The diagnostic performances of two biomarkers were considered clinically equivalent when the range of 95% confidence intervals (CIs) of the mean difference included zero. Superiority was considered when the range of 95% CI did not include zero and favored the plasma biomarker. In classification of Aβ PET status in the entire BioFINDER-2 cohort, plasma %p-tau217 had very high performance (AUC = 0.97, 95% CI: 0.95, 0.98), which was clinically equivalent to that of CSF Elecsys p-tau181/Aβ42 (AUC = 0.97, 95% CI: 0.96, 0.98) or CSF Elecsys Aβ42/40 (AUC = 0.96, 95% CI: 0.95, 0.97) (Fig. 1a and Extended Data Table 1). Similar results were obtained for classification of Aβ PET status in the entire Knight ADRC cohort: plasma %p-tau217 had an AUC (0.97, 95% CI: 0.95, 0.99) that was clinically equivalent to CSF Lumipulse Aβ42/40 (AUC = 0.96, 95% CI: 0.94, 0.98) and CSF Lumipulse p-tau181/Aβ42 (AUC = 0.97, 95% CI: 0.96, 0.99) (Fig. 1b). The AUCs were similar when cognitively impaired and cognitively unimpaired groups were analyzed separately (Fig. 1a,b and Extended Data Table 1). Differences between the AUCs of plasma %p-tau217 and CSF biomarker ratios are shown in Fig. 1c and Extended Data Table 1.

In classification of tau PET status in the entire BioFINDER-2 cohort, plasma %p-tau217 had very high performance (AUC = 0.95, 95% CI: 0.94, 0.97), which was superior to CSF Elecsys p-tau181/Aβ42 (AUC = 0.93, 95% CI: 0.92, 0.95) and CSF Elecsys Aβ42/40 (AUC = 0.88, 95% CI: 0.86, 0.90) (Fig. 1c). Similar results were obtained in the entire Knight ADRC cohort: plasma %p-tau217 had a higher AUC (0.98, 95% CI: 0.97, 0.99) compared to CSF Lumipulse p-tau181/Aβ42 (AUC = 0.96; 95% CI: 0.94, 0.98) or CSF Lumipulse Aβ42/40 (AUC = 0.90; 95% CI: 0.87, 0.94) (Fig. 1d). The AUCs were similar when cognitively impaired and cognitively unimpaired groups were analyzed separately (Fig. 1c,d and Extended Data Table 1).

### Accuracy and predictive value of fluid biomarkers

**Fig. 2:**
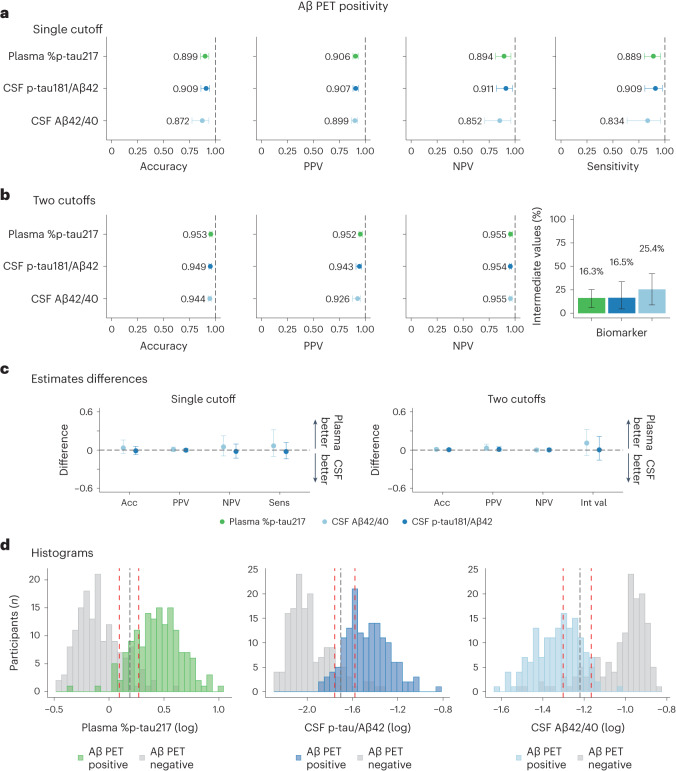
Comparison among fluid biomarkers on predicting Aβ PET positivity in cognitively impaired patients of the BioFINDER-2 cohort. a,**b**, Prediction of Aβ PET positivity in cognitively impaired participants (*n* = 304) from the BioFINDER-2 cohort, using a single-cutoff (**a**) and a two-cutoffs (**b**) approach, respectively. In the first approach, the threshold was calculated, maximizing sensitivity and fixing specificity at 90%. In the second approach, the lower threshold was obtained by maximizing specificity with sensitivity fixed at 95%, whereas the upper threshold was obtained by maximizing sensitivity while fixing specificity at 95%. Participants who fall between these two cutoffs were classified in the intermediate group. Dots and error bars represent the actual statistic and 95% CI (from bootstrapped *n* = 1,000 samples with replacement), respectively. **c**, Bootstrapped differences (*n* = 1,000 resamples with replacement stratifying by the output) between the statistics using plasma %p-tau217 (reference) and CSF biomarkers are shown in **c** for both single cutoff and two cutoffs. The horizontal dashed line is plotted at zero, representing the lack of difference between plasma and CSF biomarkers. We considered plasma and CSF biomarkers clinically equivalent if the 95% CI of the mean difference included zero and clinically superior if it did not include zero and favored plasma (>0). Differences in the number of participants in the intermediate group were scaled to a maximum of 1 to be comparable with the other differences. Dots and error bars represent the mean and 95% CI estimate from a bootstrapped sample. Vertical dashed lines represent the maximal statistical value possible (1). For the intermediate value plots, colored bars represent the actual percentage and the error bar the 95% CI. **d**, Histograms represent the distribution of the data colored by the imaging biomarker status. The vertical black line represents the threshold derived from the first approach (**a**), and red lines represent the lower and upper thresholds from the second approach (**b**). Aβ PET positivity was assessed as Centiloids ≥ 37. CSF, cerebrospinal fluid; CI, confidence interval; NPV, negative predictive value; PPV, positive predictive value.

**Extended Data Fig. 1:**
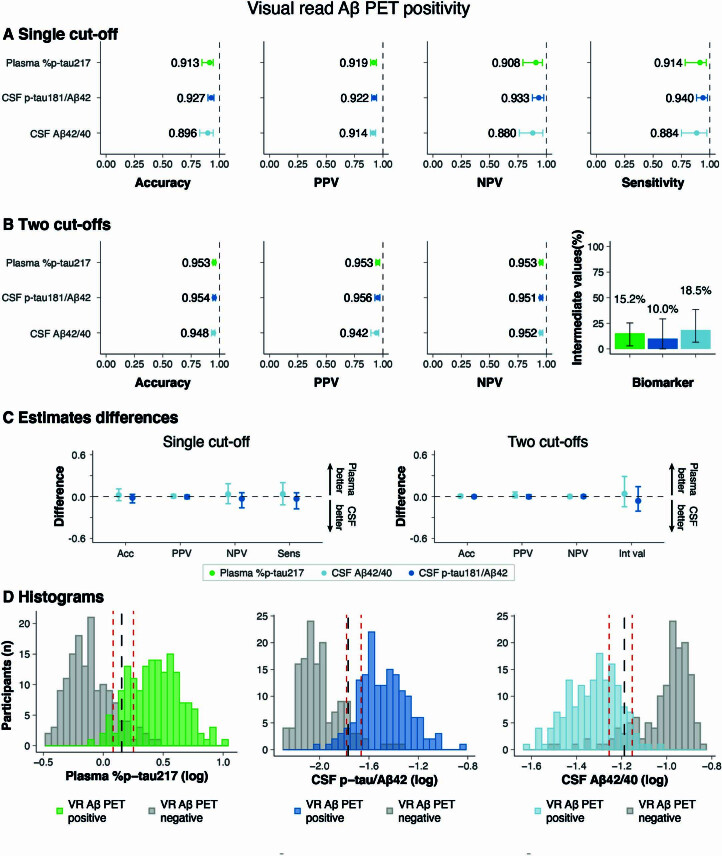
Comparison among fluid biomarkers on predicting Aβ PET visual read positivity in cognitively impaired patients of the BioFINDER-2 cohort with in-bag estimates. Prediction of Aβ PET visual read positivity in cognitively impaired participants from the BioFINDER-2 cohort, using a single cut-off (**a**) and two cut-offs (**b**) approaches, respectively. In the first approach, the cut-off was calculated maximizing sensitivity fixing specificity at 90%. In the second approach, the lower cut-off was obtained by maximizing specificity with sensitivity fixed at 95%, whereas the upper cut-off was obtained by maximizing sensitivity fixing specificity at 95%. Participants that fall between these two cut-offs were classified in the intermediate group. Dots and error bars represent the actual statistic and 95%CI, respectively. Bootstrapped differences (n = 1,000 resamples with replacement stratifying by the output) between the statistics using plasma %p-tau217 (reference) and CSF biomarkers are shown in (**c**) for both single and two cut-offs. A horizontal dashed line is plotted at zero representing the lack of difference between plasma and CSF biomarkers. We considered plasma and CSF biomarkers clinically equivalent if the 95%CI of the mean difference included zero and clinically superior if it did not include zero and favored plasma (>0). Differences in number of participants in the intermediate group have been scaled to a maximum of one to be comparable to the other differences. Dots and error bars represent the mean and 95%CI estimate from a bootstrapped sample. Vertical dashed lines represent the maximal statistical value possible (1). For the intermediate values plots, coloured bars represent the actual percentage and error bar the 95%CI. Histograms (**d**) represent the distribution of the data coloured by the imaging biomarker status (coloured represent the positive group). Vertical black line represents the cut-off derived from the first approach (**a**), and red lines represent the lower and upper cut-offs from the second approach (B). Abbreviations: Aβ, amyloid-β, CI, confidence interval; CSF, cerebrospinal fluid; NPV, negative predictive value; PPV, positive predictive value.

Next, we focused on individuals with cognitive impairment (either MCI or dementia) who could be candidates for anti-Aβ immunotherapies if amyloid biomarker testing were positive. We evaluated clinically relevant diagnostic metrics for plasma %p-tau217, CSF p-tau181/Aβ42 and CSF Aβ42/40 when using a cutoff resulting in a specificity of 90% for Aβ PET status (≥37 Centiloids). In the BioFINDER-2 cohort, we found that plasma %p-tau217 predicted Aβ PET status with an overall accuracy of 90% (95% CI: 86%, 93%), a positive predictive value (PPV) of 91% (95% CI: 88%, 93%) and a negative predictive value (NPV) of 89% (95% CI: 81%, 96%). Notably, the performance of plasma %p-tau217 in prediction of Aβ PET status was not different from CSF Elecsys p-tau181/Aβ42 (accuracy, 91% (95% CI: 86%, 94%); PPV, 91% (95% CI: 88%, 93%); NPV, 91% (95% CI: 82%, 97%)) and CSF Elecsys Aβ42/40 (accuracy, 87% (95% CI: 77%, 93%); PPV, 90% (95% CI: 87%, 93%); NPV, 85% (95% CI: 71%, 96%)) (Fig. 2a and Table 2). Similar results were obtained when using clinical visual reads to determine Aβ PET status (Extended Data Fig. 1a and Supplementary Table 1). Similar results were also found in the Knight ADRC cohort, where plasma %p-tau217 had an overall accuracy of 94% (95% CI: 72%, 100%), a PPV of 99% (95% CI: 97%, 100%) and an NPV of 89% (95% CI: 48%, 100%), which was clinically equivalent to the performances of FDA-approved CSF Lumipulse Aβ42/40 (accuracy, 78% (95% CI: 44%, 98%); PPV, 98% (95% CI: 96%, 100%); NPV, 62% (95% CI: 32%, 100%)) and CSF Lumipulse p-tau181/Aβ42 (accuracy, 91% (95% CI: 68%, 100%); PPV, 99% (95% CI: 97%, 100%); NPV, 82% (95% CI: 45%, 100%)) (Supplementary Fig. 1a and Supplementary Table 2).

**Table 2:** Comparison among fluid biomarkers on predicting Aβ positivity in cognitively impaired BioFINDER-2 patients with in-bag estimates

Single-cutoff approach								
	Accuracy		PPV		NPV		Sensitivity	
	Mean	Difference	Mean	Difference	Mean	Difference	Mean	Difference
Plasma %p-tau217	0.90 (0.86, 0.93)	Ref.	0.91 (0.88, 0.93)	Ref.	0.89 (0.81, 0.96)	Ref.	0.89 (0.80, 0.96)	Ref.
CSF p-tau/Aβ42	0.91 (0.86, 0.94)	−0.01 (−0.06, 0.05)	0.91 (0.88, 0.93)	0.00 (−0.02, 0.02)	0.91 (0.82, 0.97)	−0.02 (−0.10, 0.08)	0.91 (0.81, 0.97)	−0.02 (−0.12, 0.10)
CSF Aβ42/40	0.87 (0.77, 0.93)	0.03 (−0.04, 0.13)	0.90 (0.87, 0.93)	0.01 (−0.02, 0.04)	0.85 (0.71, 0.96)	0.04 (−0.08, 0.19)	0.83 (0.64, 0.96)	0.05 (−0.09, 0.26)

Two-cutoffs approach								
	Accuracy		PPV		NPV		Number of intermediate participants	
	Mean	Difference	Mean	Difference	Mean	Difference	Mean	Difference*
Plasma %p-tau217	0.95 (0.94, 0.97)	Ref.	0.95 (0.94, 0.97)	Ref.	0.96 (0.94, 0.98)	Ref.	16.3 (5.9, 25.3)	Ref.
CSF p-tau/Aβ42	0.95 (0.94, 0.96)	0.00 (−0.01, 0.02)	0.94 (0.91, 0.96)	0.01 (−0.02, 0.04)	0.95 (0.95, 0.97)	0.00 (−0.02, 0.03)	16.5 (4.6, 33.6)	0.00 (−0.13, 0.18)
CSF Aβ42/40	0.94 (0.93, 0.96)	0.01 (−0.01, 0.03)	0.93 (0.88, 0.95)	0.03 (−0.01, 0.08)	0.95 (0.95, 0.97)	0.00 (−0.02, 0.02)	25.4 (8.9, 42.1)	0.09 (−0.07, 0.27)

Comparison estimates among fluid biomarkers on predicting Aβ PET positivity in BioFINDER-2 cognitively impaired individuals. For the single-cutoff approach, cutoffs of fluid biomarkers were derived by maximizing sensitivity and fixing specificity at 90% against each imaging outcome. For the two-cutoffs approach, the lower cutoff was obtained by maximizing specificity with sensitivity fixed at 95%, whereas the upper cutoff was obtained by maximizing sensitivity and fixing specificity at 95%. Participants who fall between these two cutoffs were classified in the intermediate group. Differences between the statistics using plasma %p-tau217 (reference) and CSF biomarkers are shown together with the mean values. We considered plasma and CSF biomarkers clinically equivalent if the 95% CI of the mean difference included zero and clinically superior if it did not include zero and favored plasma (>0). *Differences in the number of participants in the intermediate group were scaled to a maximum of 1 to be comparable with the other differences. Aβ PET positivity was assessed as Centiloids ≥ 37. CSF, cerebrospinal fluid; NPV, negative predictive value; PPV, positive predictive value; CI, confidence interval.

**Fig. 3:**
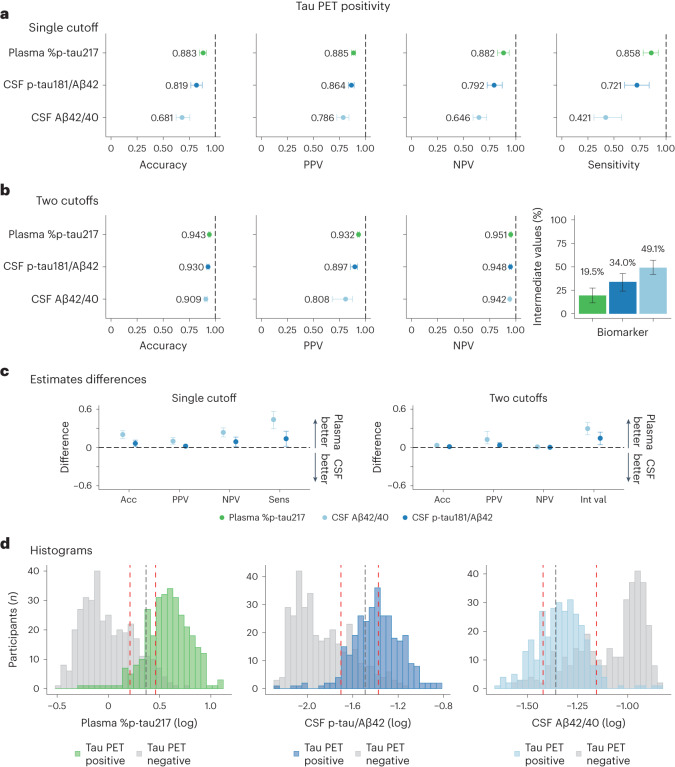
Comparison among fluid biomarkers on predicting tau PET positivity in cognitively impaired patients of the BioFINDER-2 cohort. **a**,**b**, Prediction of tau PET positivity in cognitively impaired participants from the BioFINDER-2 cohort (*n* = 663), using a single-cutoff (**a**) and a two-cutoffs (**b**) approach, respectively. In the first approach, the threshold was calculated, maximizing sensitivity and fixing specificity at 90%. In the second approach, the lower threshold was obtained by maximizing specificity with sensitivity fixed at 95%, whereas the upper threshold was obtained by maximizing sensitivity and fixing specificity at 95%. Participants who fall between these two cutoffs were classified in the intermediate group. Dots and error bars represent the actual statistic and 95% CI, respectively. Vertical dashed lines represent the maximal statistical value possible (1). For the intermediate value plots, colored bars represent the actual percentage and the error bar the 95% CI. **c**, Bootstrapped differences (*n* = 1,000 resamples with replacement stratifying by the output) between the statistics using plasma %p-tau217 (reference) and CSF biomarkers are shown in **c** for both single cutoff and two cutoffs. The horizontal dashed line is plotted at zero, representing the lack of difference between plasma and CSF biomarkers. We considered plasma and CSF biomarkers clinically equivalent if the 95% CI of the mean difference included zero. Differences in the number of participants in the intermediate group were scaled to a maximum of 1 to be comparable with the other differences. Dots and error bars represent the mean and 95% CI estimate from a bootstrapped sample. **d**, Histograms represent the distribution of the data colored by the imaging biomarker status. The vertical black line represents the threshold derived from the first approach (**a**), and red lines represent the lower and upper thresholds from the second approach (**b**). Tau PET positivity was assessed using an in-house previously validated threshold (SUVR > 1.32). Three individuals were excluded from the histograms in **d** (only for visualization purposes) due to very low values of plasma %p-tau217. CSF, cerebrospinal fluid; CI, confidence interval; NPV, negative predictive value; PPV, positive predictive value; SUVR, standardized uptake value ratio.

When predicting tau PET status in cognitively impaired patients in the BioFINDER-2 cohort, plasma %p-tau217 had an overall accuracy of 88% (95% CI: 85%, 91%), a PPV of 88% (95% CI: 86%, 90%) and an NPV of 88% (95% CI: 82%, 94%), which was superior to the performance of CSF Elecsys p-tau181/Aβ42 (accuracy, 82% (95% CI: 76%, 87%); PPV, 86% (95% CI: 83%, 89%); NPV, 79% (95% CI: 72%, 87%)) and CSF Elecsys Aβ42/40 (accuracy, 68% (95% CI: 62%, 76%); PPV, 79% (95% CI: 73%, 84%); NPV, 65% (95% CI: 59%, 72%)) (Fig. 3a,c and Table 3). In the Knight ADRC cohort, the diagnostic metrics of plasma %p-tau217 were clinically equivalent to those of the CSF measures (Supplementary Fig. 2a,c and Supplementary Table 3).

**Table 3:** Comparison among fluid biomarkers on predicting tau PET positivity in cognitively impaired patients with in-bag estimates

Single-cutoff approach								
	Accuracy		PPV		NPV		Sensitivity	
	Mean	Difference	Mean	Difference	Mean	Difference	Mean	Difference
Plasma %p-tau217	0.88 (0.85, 0.91)	Ref.	0.88 (0.86, 0.90)	Ref.	0.88 (0.82, 0.94)	Ref.	0.86 (0.78, 0.93)	Ref.
CSF p-tau/Aβ42	0.82 (0.76, 0.87)	0.06 (0.01, 0.12)	0.86 (0.83, 0.89)	0.02 (0.00, 0.04)	0.79 (0.72, 0.87)	0.09 (0.01, 0.16)	0.72 (0.60, 0.84)	0.14 (0.02, 0.25)
CSF Aβ42/40	0.68 (0.62, 0.76)	0.20 (0.14, 0.26)	0.79 (0.73, 0.84)	0.10 (0.05, 0.15)	0.65 (0.59, 0.72)	0.24 (0.16, 0.31)	0.42 (0.31, 0.57)	0.44 (0.29, 0.56)

Two-cutoffs approach								
	Accuracy		PPV		NPV		Number of intermediate participants	
	Mean	Difference	Mean	Difference	Mean	Difference	Mean	Difference*
Plasma %p-tau217	0.94 (0.94, 0.95)	Ref.	0.93 (0.92, 0.94)	Ref.	0.95 (0.94, 0.96)	Ref.	19.5 (11.6, 27.5)	Ref.
CSF p-tau/Aβ42	0.93 (0.92, 0.94)	0.01 (0.00, 0.02)	0.90 (0.85, 0.92)	0.04 (0.01, 0.08)	0.95 (0.94, 0.96)	0.00 (−0.01, 0.01)	34.0 (24.1, 42.8)	0.14 (0.04, 0.24)
CSF Aβ42/40	0.91 (0.89, 0.92)	0.03 (0.02, 0.05)	0.81 (0.68, 0.88)	0.12 (0.06, 0.25)	0.94 (0.94, 0.95)	0.01 (0.00, 0.02)	49.1 (41.9, 57.0)	0.30 (0.20, 0.39)

Comparison estimates among fluid biomarkers on predicting tau PET positivity in cognitively impaired patients from the BioFINDER-2 cohort. For the single-cutoff approach, the cutoffs of fluid biomarkers were derived by maximizing sensitivity and fixing specificity at 90% against each imaging outcome. For the two-cutoffs approach, the lower cutoff was obtained by maximizing specificity with sensitivity fixed at 95%, whereas the upper cutoff was obtained by maximizing sensitivity and fixing specificity at 95%. Participants who fall between these two cutoffs were classified in the intermediate group. Differences between the statistics using plasma %p-tau217 (reference) and CSF biomarkers are shown together with the mean values. We considered plasma and CSF biomarkers clinically equivalent if the 95% CI of the mean difference included zero and clinically superior if it did not include zero and favored plasma (>0). *Differences in the number of participants in the intermediate group were scaled to a maximum of 1 to be comparable with the other differences. Tau PET positivity was assessed using an in-house previously validated cutoff (SUVR > 1.32 for both cohorts in Braak I–IV). CSF, cerebrospinal fluid; NPV, negative predictive value; PPV, positive predictive value; SUVR, standardized uptake value ratio; CI, confidence interval

### Use of a two-cutoffs approach to improve diagnostic accuracy

We also evaluated for potential improvements in diagnostic accuracy by applying an approach with two cutoffs, which divides results into three categories: those with clearly normal values, those with clearly abnormal values and those with intermediate values. The upper cutoff was set at a value yielding a specificity of 95%, while maximizing sensitivity, and the lower cutoff was set at a value resulting in a sensitivity of 95%, while maximizing specificity. When the two-cutoffs approach was applied to predict Aβ PET positivity in cognitively impaired patients in the BioFINDER-2 cohort, plasma %p-tau217 had an overall accuracy of 95% (95% CI: 94%, 97%), a PPV of 95% (95% CI: 94%, 97%) and an NPV of 96% (95% CI: 94%, 98%), which were clinically equivalent to the performances of CSF Elecsys p-tau181/Aβ42 (accuracy, 95% (95% CI: 94%, 96%); PPV, 94% (95% CI: 91%, 96%); NPV, 95% (95% CI: 95%, 97%)) and CSF Elecsys Aβ42/40 (accuracy, 94% (95% CI: 93%, 96%); PPV, 93% (95% CI: 88%, 95%); NPV, 95% (95% CI: 95%, 97%)) (Fig. 2b and Table 2). The percentage of individuals with intermediate values was 16% (95% CI: 6%, 25%) for plasma %p-tau217, 17% (95% CI: 5%, 34%) for CSF Elecsys p-tau181/Aβ42 and 25% (95% CI: 9%, 42%) for CSF Elecsys Aβ42/40 (Fig. 2b). Similar results were obtained when FDA-approved visual reads were used to determine the Aβ PET status (Extended Data Fig. 1b and Supplementary Table 1) and in the Knight ADRC cohort (Supplementary Fig. 1b and Supplementary Table 2).

When predicting tau PET status in cognitively impaired individuals in the BioFINDER-2 cohort using the two-cutoffs approach, we found that plasma %p-tau217 had an overall accuracy of 94% (95% CI: 94%, 95%), a PPV of 93% (95% CI: 92%, 94%) and an NPV of 95% (95% CI: 94%, 96%), which was superior to the performances of CSF Elecsys p-tau181/Aβ42 (accuracy, 93% (95% CI: 92%, 94%); PPV, 90% (95% CI: 85%, 92%); NPV, 95% (95% CI: 94%, 96%)) and CSF Elecsys Aβ42/40 (accuracy, 91% (95% CI: 89%, 92%); PPV, 0.81% (95% CI: 68%, 88%); NPV, 94% (95% CI: 94%, 95%)) (Fig. 3b and Table 3). The percentage of individuals with intermediate values was lower for plasma %p-tau217 (20%, 95% CI: 12%, 28%) compared to those for CSF Elecsys p-tau181/Aβ42 (34%, 95% CI: 24%, 43%) and for CSF Elecsys Aβ42/40 (49%, 95% CI: 42%, 57%) (Fig. 3b). The results obtained in Knight ADRC showed a similar performance between plasma and CSF biomarkers (Supplementary Fig. 2b and Supplementary Table 3).

**Extended Data Fig. 2:**
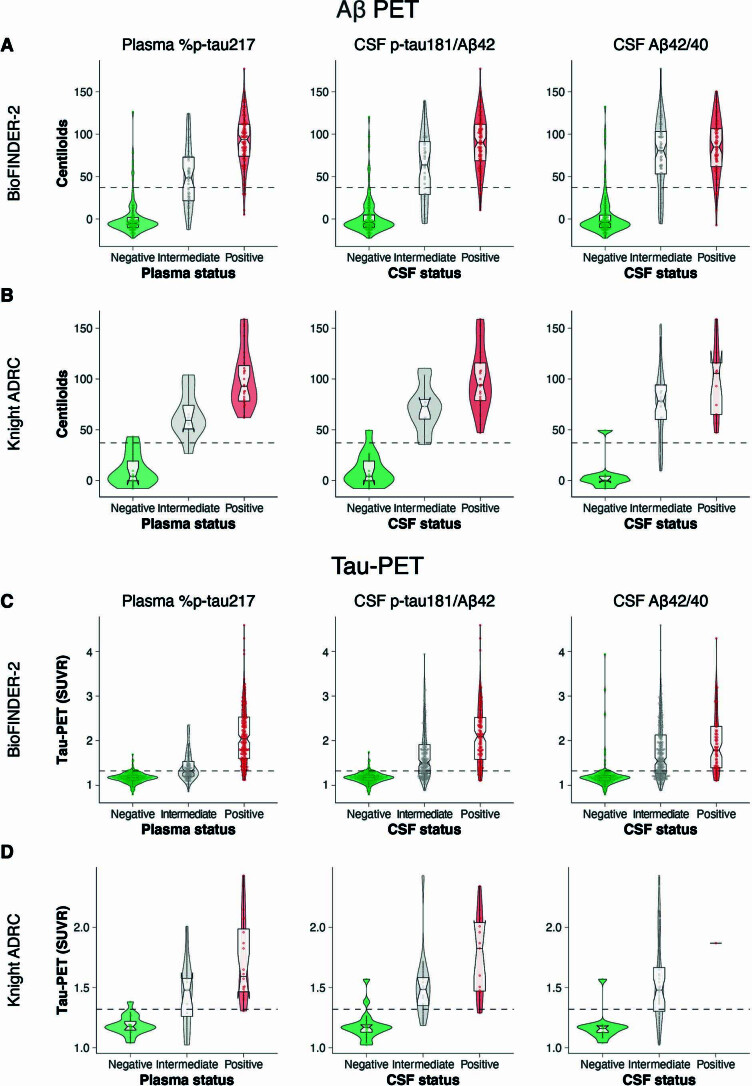
Continuous Aβ and tau PET measures by categorized fluid biomarkers groups. Comparison between categorised fluid biomarkers levels and continuous measures of Aβ- (Centiloids, **a, b**) and tau PET (SUVR, **c, d**) quantification. Fluid biomarkers were categorised using the two-cut-off approach. The lower cut-off was obtained by maximizing specificity with sensitivity fixed at 95%, whereas the upper cut-off was obtained by maximizing sensitivity fixing specificity at 95%. Participants that fall between these two cut-offs were classified in the intermediate group. Dots represent individual participants. In all cases, central band of the boxplot represents the median of the group, the lower and upper hinges correspond to the first and third quartiles, and the whiskers represent the maximum/minimum value or the 1.5 IQR from the hinge, whatever is lower. Horizontal dashed lines represent the cut-off of positivity for each imaging marker (Aβ PET: ≥37 Centiloids, Tau PET: >1.32 SUVR for both cohorts). Abbreviations: Aβ, amyloid-β; CI, confidence interval; CSF, cerebrospinal fluid; IQR, inter-quantile range; NPV, negative predictive value; PPV, positive predictive value; SUVR, standardized uptake value ratio.

We investigated whether the groups with intermediate fluid biomarker values also had intermediate values for the reference standard—that is, Aβ PET Centiloids or tau PET SUVR. We found that individuals with intermediate plasma %p-tau217 values had values for Aβ PET and tau PET that were near the cutoffs for abnormality (Extended Data Fig. 2). Additionally, the group with intermediate plasma %p-tau217 values had Aβ PET and tau PET values that were higher than the normal plasma %p-tau217 group and lower than the abnormal plasma %p-tau217 group (*P* < 0.001 in all cases). In the BioFINDER-2 cohort, the mean (s.d.) Centiloids was 0.4 (20.3) for the %p-tau217 negative group, 49.1 (36.5) for the %p-tau217 intermediate group and 91.4 (30.1) for the %p-tau217 positive group.

### Comparison to a clinical AD diagnosis

Finally, we examined the accuracy of plasma %p-tau217 for clinical diagnosis of symptomatic AD versus other neurodegenerative diseases. This diagnosis was made based on clinical symptoms assessed by a dementia specialist and included consideration of AD biomarker testing by either CSF or Aβ PET. It is important to highlight that, if the clinical symptoms were not related to AD, the participant was classified in the other neurodegenerative diseases group even with positive AD biomarkers, as these results may indicate concomitant AD pathology. A description of specific diagnosis for the cognitively impaired participants is shown in Supplementary Table 4. In cognitively impaired individuals in the BioFINDER-2 cohort, we found that blood plasma %p-tau217 exhibited an AUC of 0.94 (95% CI: 0.92, 0.96) in distinguishing individuals with and without symptomatic AD (Supplementary Table 5), which was clinically equivalent to CSF p-tau181/Aβ42 (95%, 95% CI: 93%, 96%) and CSF Aβ42/40 (93%, 95% CI: 91%, 95%). Furthermore, plasma %p-tau217 had an overall accuracy of 86% (95% CI: 82%, 89%), a PPV of 89% (95% CI: 87%, 91%) and an NPV of 84% (95% CI: 77%, 89%) (Supplementary Table 6). Applying the two-cutoffs approach increased the diagnostic metrics to 93–94%, with 24% of the participants in the intermediate group (Supplementary Table 6).

### Sensitivity analyses

Several sensitivity analyses were performed to support the results reported above. First, we assessed out-of-bag statistics in the BioFINDER-2 cohort for Aβ and tau PET positivity, in which the cutoffs and the statistics were derived in different individuals from the same cohort. These results were in line with the previous analyses, showing that plasma %p-tau217 was clinically equivalent to CSF biomarkers for predicting Aβ PET positivity using a single-cutoff approach (Supplementary Fig. 3a and Supplementary Table 7) and a two-cutoffs approach (Supplementary Fig. 3b and Supplementary Table 8). For tau PET, we generally observed higher estimates of plasma %p-tau217 compared to the two CSF biomarkers (Supplementary Fig. 4 and Supplementary Tables 7 and 8).

**Extended Data Fig. 3:**
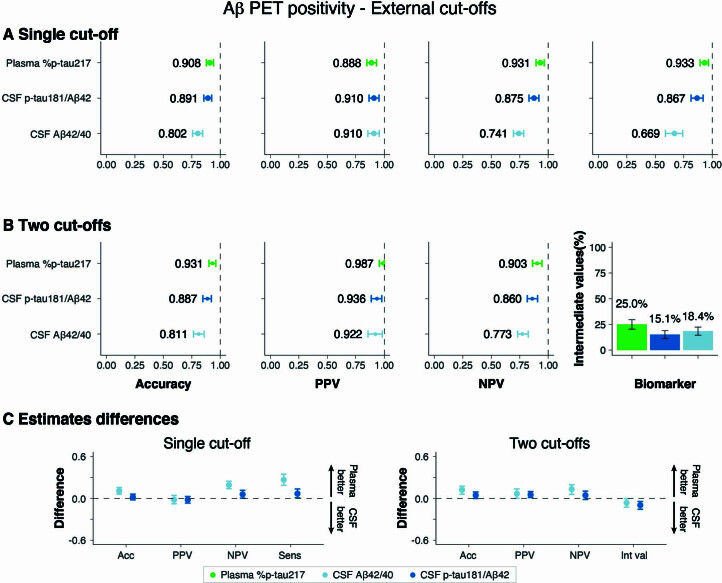
Comparison among fluid biomarkers on predicting Aβ PET positivity in cognitively impaired patients of the BioFINDER-2 cohort using external cut-offs. Prediction of Aβ PET positivity in cognitively impaired participants from the BioFINDER-2 cohort, using a single cut-off (**a**) and two cut-offs (**b**) approaches, respectively. In the first approach, the cut-off was calculated maximizing sensitivity fixing specificity at 90%. In the second approach, the lower cut-off was obtained by maximizing specificity with sensitivity fixed at 95%, whereas the upper cut-off was obtained by maximizing sensitivity fixing specificity at 95%. Participants that fall between these two cut-offs were classified in the intermediate group. Dots and error bars represent the actual statistic and 95%CI, respectively. The external cut-off method derives the cut-offs in independent cohorts. Plasma %p-tau217 cut-offs were derived in the Knight ADRC cohort, and CSF biomarkers were derived in the UCSF cohort. Bootstrapped differences (n = 1,000 resamples with replacement stratifying by the output) between the statistics using plasma %p-tau217 (reference) and CSF biomarkers are shown in (c) for both single and two cut-offs. A horizontal dashed line is plotted at zero representing the lack of difference between plasma and CSF biomarkers. We considered plasma and CSF biomarkers clinically equivalent if the 95%CI of the mean difference included zero and clinically superior if it did not include zero and favored plasma (>0). Differences in number of participants in the intermediate group have been scaled to a maximum of one to be comparable to the other differences. Dots and error bars represent the mean and 95%CI estimate from a bootstrapped sample. Vertical dashed lines represent the maximal statistical value possible (1). For the intermediate values plots, coloured bars represent the actual percentage and error bar the 95%CI. Aβ PET positivity was assessed as Centiloids≥37. Abbreviations: Aβ, amyloid-β, CI, confidence interval; CSF, cerebrospinal fluid; NPV, negative predictive value; PPV, positive predictive value.

Second, we derived fluid biomarker cutoffs in independent cohorts and tested them in BioFINDER-2 participants. Plasma %p-tau217 cutoffs were derived in Knight ADRC participants and CSF biomarker cutoffs in participants from the University of California, San Francisco (UCSF) (Supplementary Methods). The obtained results were similar to those detailed in the previous sections. In brief, the performances of plasma %p-tau217 were clinically equivalent to or slightly higher than those of CSF biomarkers when using both the single-cutoff approach (Extended Data Fig. 3a and Supplementary Table 7) and the two-cutoffs approach (Extended Data Fig. 3b and Supplementary Table 8) for prediction of Aβ positivity.

Additionally, we examined whether the use of plasma p-tau217 as predictor with non-phosphorylated tau as covariate (rather than the ratio of p-tau217/non-phosphorylated tau (%p-tau217)) resulted in any significant change in our results. In summary, the differences between these two approaches were very small, as can be observed in Supplementary Figs. 5 and 6 and in Supplementary Tables 9 and 10.

Finally, we also tested the consistency across time of our results in a subcohort of 40 Knight ADRC participants with available longitudinal plasma %p-tau217 measures (mean (s.d.) time = 3.03 (0.65) years). Only one (2.5%) of these participants changed %ptau217 biomarker status during follow-up testing, supporting the consistency of plasma %p-tau217 measures when plasma sampling and %ptau217 testing is repeated (Supplementary Fig. 7).

## Discussion

The major finding of this study was that plasma %p-tau217 classifies both Aβ and tau PET status with very high accuracy (AUCs of 0.96 and 0.98) across two independent cohorts. When compared to clinically used and FDA-approved CSF tests, the performance of plasma %p-tau217 was clinically equivalent in classification of Aβ PET status and was superior in classification of tau PET status. Notably, in the cognitively impaired subcohorts, the PPV of plasma %p-tau217 was equivalent to the CSF tests, demonstrating that the blood test could confirm the presence of Aβ pathology as accurately as CSF tests. A blood test with such high performance could replace CSF testing or Aβ PET when determining the presence of brain Aβ pathology in patients with cognitive symptoms. Given the widespread acceptance and accessibility of blood collection, high-performance blood tests could enable AD biomarker testing on a greater scale than is currently possible and to a much broader population, thereby enabling more accurate diagnosis of AD worldwide.

In patients with MCI and mild dementia who may be candidates for anti-amyloid treatments, plasma %p-tau217 classified Aβ PET status with an accuracy, a PPV and an NPV of approximately 90% when a standard approach using a single cutoff was applied. Accuracies of 90–95% are considered excellent or outstanding for the detection of pathology and match or exceed clinically used CSF tests. For instance, the FDA-approved Elecsys CSF p-tau181/Aβ42 test has, in previous studies, classified Aβ PET status with overall accuracies of 89–90% (refs. ^39–41^), which was replicated in the present study. The performance of the FDA-approved Lumipulse CSF Aβ42/40 test is more complex to evaluate because different approaches have been applied, including using two cutoffs^42,43^, but in one large study the test classified Aβ PET status with an AUC of 0.97 (ref. ^44^). Notably, Aβ PET and tau PET are not perfectly accurate in detection of neuropathology^45,46^, and, in the small proportion of cases that have discordant CSF and PET results, it is not clear whether this is due to inaccuracy of CSF or PET measures. Given some imprecision in the reference standard for amyloid positivity, FDA-appproved CSF assays as well as plasma %p-tau217 may be performing at the maximum level that is achievable.

Plasma %p-tau217 also correctly classified Aβ PET positivity status for cognitively unimpaired participants with AUCs of 0.96 in both BioFINDER-2 and Knight ADRC. This is also consistent with a recent report from the AHEAD 3–45 study^47^ supporting the utility of plasma %p-tau217 as a screening test for preclinical AD using a similar mass spectrometry platform. With such high performance, these blood tests have the potential to support Aβ pathology identification among preclinical populations and in participant recruitment for preventive trials assessing anti-amyloid drugs. Detection of Aβ positivity using mass spectrometry %p-tau217 in cognitively normal cohorts appears better than what has been reported when using plasma p-tau217 immunoassays, although this must be confirmed in head-to-head studies^22,23,48–50^.

In this study, we used Centiloids ≥ 37 as the primary measure of Aβ PET positivity based on the inclusion criteria of recent clinical trials for donanemab^7^. Given that Aβ PET status is normally assessed by visual assessment in clinical care, and the FDA and the European Medicines Agency (EMA) have approved visual reads of Aβ PET, we also included visual read as an additional outcome in the main cohort. The obtained results were very similar for both Aβ PET outcomes, demonstrating very high accuracy of plasma %p-tau217 for detecting Aβ pathology, which was clinically equivalent to that of CSF biomarkers. Notably, there was very high agreement between quantitative and visual read for Aβ PET status in our cohort (~95%), consistent with previous studies showing very high agreement between visual assessment and Aβ PET quantification^45,51–54^.

In addition to highly accurate classification of Aβ PET status, plasma %p-tau217 classified tau PET status with an overall accuracy, a PPV and an NPV of 87–88% in the cognitively impaired group of the main cohort. The CSF assays were also able to classify tau PET status but were inferior to plasma %p-tau217. Because tau PET is an excellent indicator of symptomatic AD^5^, the superior classification of tau PET status by plasma %p-tau217 suggests that this measure may have additional value in determining whether cognitive impairment is likely to be due to AD. Overall, the high performance of plasma %p-tau217 in classifying Aβ and tau PET status indicates that this BBM may be able to replace approved CSF and PET measures in the diagnostic workup of AD.

As expected, the performance of plasma %p-tau217 improved after applying an approach using two cutoffs to categorize individuals as positive, negative or intermediate. Use of this approach for plasma %p-tau217 resulted in a PPV and an NPV of 95% for Aβ PET status with fewer than 20% of participants in the intermediate zone, which was clinically equivalent to the CSF assays. Notably, individuals with intermediate values of plasma %p-tau217 also had Aβ PET values close to the threshold used to determine Aβ PET status: they have borderline values across multiple modalities, indicating that they may have early AD brain pathological changes. For a more definitive result, these individuals could either repeat the same test at a later time or undergo testing with another type of diagnostic test (for example, PET or CSF). Notably, the two-cutoffs approach is currently employed for the FDA-approved CSF Lumipulse test^42,43^ and has been suggested for AD BBMs^17,55^, especially when very high accuracy is needed. Very high confidence in Aβ status is especially important for patients who might be eligible for anti-amyloid immunotherapies, especially given the high costs associated with such therapies as well as the clinical resources required, including repeated infusions and magnetic resonance imaging scans. Tests with a PPV of at least 95% would be preferable so that fewer than 5% of patients receiving treatment would be amyloid negative. Such an approach using two cutoffs could also enable much faster and less expensive enrollment of participants into clinical trials because Aβ status could be determined using plasma %p-tau217 alone for the large majority of individuals^56^.

The main strength of this study includes the use of a high-performance plasma %p-tau217 assay in combination with clinically used CSF and Aβ and tau PET biomarkers across two large and well-phenotyped cohorts. We also reported PPV and NPV estimates, in addition to sensitivity, as they are more clinically informative. Nonetheless, we acknowledge that these measures are influenced by the prevalence of the disease or pathology detected. In the present study, the Aβ positivity ranged between 50% and 74% in the two cognitively impaired populations, which agrees with most other memory clinic cohorts of patients with MCI or mild dementia. For example, in the large-scale IDEAS study, 55% of MCI and 70% of dementia cases were amyloid positive^12^. Limitations include the relatively few individuals in the Knight ADRC cohort with cognitive impairment and the lack of a sufficiently large group of individuals with both antemortem biomarker and postmortem data available. In addition, although hundreds of millions of mass spectrometry clinical tests are run every year for several clinical applications (for example, newborn screening, analysis of drugs of abuse and steroid analysis)^57^, they typically have a higher cost per assay than immunoassays, and the corresponding analytical platforms are also less widely available and require more technical and operational expertise. Nonetheless, to date, mass spectrometry measures of plasma p-tau217 have shown the best performance for assessing the presence of Aβ pathology compared to immunoassays^29^. Future head-to-head comparisons may address whether the benefits from higher accuracy provided by mass spectrometry assays outweigh the relative practicability and scalability offered by immunoassays. Finally, minoritized populations were not well enough represented in the study cohorts, even though many study participants had lower education levels and many comorbidities. Future studies should investigate the performance of plasma %p-tau217 in broader primary care–based populations.

In summary, plasma %p-tau217 can be used to determine Aβ status with a PPV and an accuracy of 95% in more than 80% of cognitively impaired patients and shows clinically equivalent or superior performance to clinically used FDA-approved CSF-based tests in classification of Aβ and tau PET status. Implementation of blood %p-tau217 in clinical practice would substantially reduce the need for PET or CSF testing, thereby enhancing access to accurate AD diagnosis in clinics worldwide, and enable determination of amyloid status in patients with MCI or mild dementia who might benefit from anti-amyloid immunotherapies.

## Methods

### Study design

This study included participants from two independent observational cohorts: the BioFINDER-2 study from Sweden and the Knight ADRC study from the United States. The Swedish BioFINDER-2 study (NCT03174938) was described previously in detail^58^. The participants were recruited at Skåne University Hospital and the Hospital of Ängelholm in Sweden (dates of enrollment: April 2017 to June 2022) and included individuals who were cognitively unimpaired (either no cognitive concerns or subjective cognitive decline (SCD)) or cognitively impaired (classified as having MCI, AD dementia or various other neurodegenerative diseases)^23^. Participants were categorized as having MCI if they performed worse than −1.5 s.d. in any cognitive domain according to age and education stratified test norms, as previously described^58^. AD dementia was diagnosed if the individual was Aβ positive by PET or CSF and met the *Diagnostic and Statistical Manual of Mental Disorders*, Fifth Edition, criteria for AD^59^. The Knight ADRC cohort was previously described and enrolls individuals into longitudinal observational research studies of memory and aging; most participants live in the greater metropolitan area of St. Louis, Missouri, USA^44^. Samples used for the current study were collected from participants between 6 February 2013 and 12 March 2020. Participants were assessed with the Clinical Dementia Rating (CDR)^60^, and individuals included in the current study were either cognitively unimpaired (CDR = 0) or cognitively impaired (CDR > 0) with a clinical syndrome typical of AD (either MCI or dementia) based on standard criteria^61^. Additionally, participants included had undergone both an Aβ PET and a tau PET scan within 2 years of CSF and had sufficient plasma available for analysis.

### Fluid biomarkers

#### CSF AD biomarker measurements

CSF samples were collected and handled according to current international recommendations^44,62^. In the Swedish BioFINDER-2 study, CSF concentrations of Aβ42 and p-tau181 were measured using Roche Elecsys CSF electrochemiluminescence immunoassays on a fully automated cobas e 601 instrument (Roche Diagnostics). Aβ40 concentrations were measured with the Roche NeuroToolKit on cobas e 411 and e 601 instruments (Roche Diagnostics). The ratio of CSF p-tau181 to Aβ42 (p-tau181/Aβ42) as measured by Elecsys assays was validated^63^ and FDA approved in December 2022 for the detection of Aβ plaques associated with AD for individuals with cognitive impairment. The Elecsys Aβ42/40 ratio was also examined. In the Knight ADRC cohort, CSF Aβ42, Aβ40 and p-tau181 concentrations were measured with an automated immunoassay platform (Lumipulse G1200, Fujirebio). The ratio of CSF Aβ42 to Aβ40 (Aβ42/40) as measured by Lumipulse assays was validated^64^ and FDA approved in May 2022 for the detection of Aβ plaques associated with AD for individuals with cognitive impairment, and, in addition, the Lumipulse Aβ42/p-tau181 ratio was also examined.

#### Blood %p-tau217 measurement

At the same session as CSF collection, blood was also collected from participants in a tube containing EDTA and centrifuged to separate plasma as previously described^65^. Blood plasma p-tau217 and non-p-tau217 were measured by liquid chromatography–tandem high-resolution mass spectrometry (LC–MS/HRMS) analysis as detailed in the Supplementary Methods. The %p-tau217 measure was calculated as the ratio of tau phosphorylated at residue 217 divided by the concentration of non-phosphorylated mid-region tau.

#### Imaging biomarker outcomes

Detailed descriptions of imaging procedures in the BioFINDER-2 and Knight ADRC cohorts were previously reported^23,66,67^. Aβ PET was performed with the EMA/FDA-approved tracer [^18^F]flutemetamol in the BioFINDER-2 cohort and with the FDA-approved tracer [^18^F]florbetapir (AV45) or [^11^C]Pittsburgh Compound B (PiB) in the Knight ADRC cohort. Mean cortical SUVR was calculated using the average signal from neocortical ROIs (bilateral orbitofrontal, medial orbitofrontal, rostral middle frontal, superior frontal, superior temporal, middle temporal and precuneus) with cerebellar gray matter as reference. SUVR values were then transformed to Centiloids, which harmonizes measures from different tracers and studies^68^. Aβ PET positivity was set at ≥37 Centiloids based on inclusion criteria in the TRAILBLAZER-ALZ studies that evaluated the clinical effects of the anti-Aβ immunotherapy donanemab^7^. Additionally, in the BioFINDER-2 study, [^18^F]flutemetamol scans were also evaluated by visual read according to an FDA-approved protocol^69^.

Tau PET scans were acquired with the [^18^F]RO948 tracer in the BioFINDER-2 cohort and with the FDA-approved [^18^F]flortaucipir tracer in the Knight ADRC cohort. These two tau PET tracers are structurally very similar and provide similar results in the cortex according to head-to-head comparisons^70^. SUVR values were calculated in a commonly used temporal meta-ROI, which includes the Braak I–IV regions and captures the regions most affected by tau, with the inferior cerebellar gray matter as reference. Previously determined thresholds were used to determine tau PET positivity (SUVR > 1.32 in both cohorts)^44,71^.

#### Endpoints

The primary outcome was the classification of amyloid pathology as determined by Aβ PET imaging. Secondary outcomes included the detection of brain tau aggregates as determined by tau PET imaging and agreement with a clinical AD diagnosis based on clinical symptoms and clinically obtained biomarker results. Main analyses were performed in cognitively impaired participants as they are the population currently eligible for anti-amyloid treatments.

#### Statistical analysis

Blood plasma %p-tau217, CSF p-tau181/Aβ42 and CSF Aβ42/40 were used as predictors in independent models. To evaluate the performance of the three fluid biomarkers in predicting the main outcomes (Aβ and tau PET status and clinical AD diagnosis), we used receiver operating characteristic (ROC) curves (*pROC* package^72^). AUCs were calculated in all participants as well as for cognitively impaired (MCI and dementia) and cognitively unimpaired (controls and SCD) subgroups. DeLong’s test included in the same R package was used to calculate mean and 95% CI differences of the plasma and CSF AUCs.

Next, we evaluated the performance of these biomarkers using only cognitively impaired participants, as this group is more relevant to the intended use of these tests in clinical practice. We used two approaches to categorize patients based on their fluid biomarkers. First, we created two groups (that is, positive and negative) based on a threshold derived by maximizing the sensitivity while fixing the specificity at 90% against each outcome independently (*cutpointr* package^73^). For this approach, we compared the accuracy, PPV, NPV and sensitivity of plasma %p-tau217 to the FDA-approved CSF biomarkers. In a second approach, we created three groups of participants (that is, positive, negative and intermediate) using two different thresholds, as recently described^17^. This was implemented independently for every outcome and cohort. The lower threshold was obtained by maximizing the specificity with the sensitivity fixed at 95%, whereas the upper threshold was obtained by the maximizing sensitivity with the specificity fixed at 95%. Participants with biomarker levels between these two thresholds were categorized as intermediate. For this approach, we compared the accuracy, PPV and NPV and the number of patients categorized as intermediate. In this approach, accuracy, PPV and NPV only took into account participants in the negative and positive groups as the intermediate group was assessed by the percentage of participants categorized on it.

Statistics were calculated as the mean of bootstrapped sample (*n* = 1,000 resamples with replacement stratifying by the output), from which we also calculated the 95% CI. The bootstrapped sample was also used to calculate the difference of all plasma %p-tau217 statistics (reference) and those from the CSF biomarkers. We considered plasma and CSF biomarkers clinically equivalent if the 95% CI of the mean difference included zero and superior if the 95% CI did not include zero while favoring plasma results.

All statistics were calculated using the same sample in which the cutoff was derived (in-bag), due to the small sample size in the replication cohort. To assess the effect of deriving the cutoff in an independent sample, we performed two sensitivity analyses in the BioFINDER-2 cohort. First, we performed the bootstrap approach as done in the *cutpointr* package^73^. This method derives the cutoffs in a bootstrapped sample (same sample size with replacement) and calculates the statistics in the individuals not included in the derivation of the cutoff. This completely independent remaining sample will include, on average, 36.8% of all individuals in the original sample when this procedure is done multiple times (*n* = 1,000 here)^74^. Second, we also derived the plasma %p-tau217 cutoffs in the Knight ADRC cohort and tested them in the BioFINDER-2 cohort (Supplementary Methods). Given that the CSF biomarkers were measured using two different FDA-approved assays in the two cohorts (Roche Elecsys in BioFINDER-2 and Fujirebio Lumipulse in Knight ADRC), we derived the CSF biomarker cutoffs for the Roche Elecsys assay in a third independent cohort from UCSF^75^ (Supplementary Methods), following the same approach.

As a sensitivity analysis, we also calculated the estimates using plasma p-tau217 as predictor while adjusting for plasma non-phosphorylated mid-region tau, instead of calculating the plasma ratio (that is, occupancy), using a logistic regression model.

All statistical analyses were performed in R version 4.1.0 (https://www.r-project.org/).

### Reporting summary

Further information on research design is available in the Nature Portfolio Reporting Summary linked to this article.

## Online content

Any methods, additional references, Nature Portfolio reporting summaries, source data, extended data, supplementary information, acknowledgements, peer review information; details of author contributions and competing interests; and statements of data and code availability are available at 10.1038/s41591-024-02869-z.

### Supplementary information

**Supplementary Results**: Supplementary Table 1: Comparison among fluid biomarkers on predicting Aβ PET visual read positivity in cognitively impaired patients with in-bag estimates. Supplementary Table 2: Comparison among fluid biomarkers on predicting Aβ positivity in cognitively impaired Knight ADRC individuals with in-bag estimates. Supplementary Fig. 1: Comparison among fluid biomarkers on predicting Aβ PET positivity in cognitively impaired patients of the Knight ADRC cohort with in-bag estimates. Supplementary Fig. 2: Comparison among fluid biomarkers on predicting tau PET positivity in cognitively impaired patients of the Knight ADRC cohort with in-bag estimates. Supplementary Table 3: Comparison among fluid biomarkers on predicting tau PET positivity in cognitively impaired patients with in-bag estimates. Supplementary Table 4: Diagnosis of cognitively impaired participants. Supplementary Table 5: Concordance of fluid biomarkers and AD diagnosis. Supplementary Table 6: Accuracy of plasma %p-tau217 in classifying diagnosis as AD or non-AD in cognitively impaired patients of the BioFINDER-2 cohort. Supplementary Table 7: Comparison among fluid biomarkers on predicting Aβ and tau PET positivity in cognitively impaired patients using a single-cutoff approach with out-of-bag estimates. Supplementary Table 8: Comparison among fluid biomarkers on predicting Aβ and tau PET positivity in cognitively impaired patients using a two-cutoffs approach with out-of-bag estimates. Supplementary Fig. 3: Comparison among fluid biomarkers on predicting Aβ PET positivity in cognitively impaired patients of the BioFINDER-2 cohort with out-of-bag statistics with the bootstrap approach. Supplementary Fig. 4: Comparison among fluid biomarkers on predicting tau PET positivity in cognitively impaired patients of the BioFINDER-2 cohort with out-of-bag statistics with the bootstrap approach. Supplementary Fig. 5: Comparison between plasma p-tau217 and %p-tau217 on predicting Aβ PET positivity in cognitively impaired patients of the BioFINDER-2 cohort with in-bag estimates. Supplementary Fig. 6: Comparison between plasma p-tau217 and %p-tau217 on predicting tau PET positivity in cognitively impaired patients of the BioFINDER-2 cohort with in-bag estimates. Supplementary Table 9: Comparison between plasma p-tau217 and %p-tau217 on predicting Aβ and tau PET positivity in cognitively impaired patients using a single-cutoff approach with in-bag estimates. Supplementary Table 10: Comparison between plasma p-tau217 and %p-tau217 on predicting Aβ and tau PET positivity in cognitively impaired patients using a two-cutoffs approach with in-bag estimates. Supplementary Fig. 7: Longitudinal trajectories of plasma %p-tau217 in Knight ADRC participants. **Supplementary Methods**: Plasma %p-tau217 analysis by immunoprecipitation mass spectrometry (IP-MS). Comparison of plasma %p-tau217 measurements in the Knight ADRC and BioFINDER-2. Description of UCSF cohort.

Reporting Summary

## Figures and Tables

**Extended Data Table 1:**
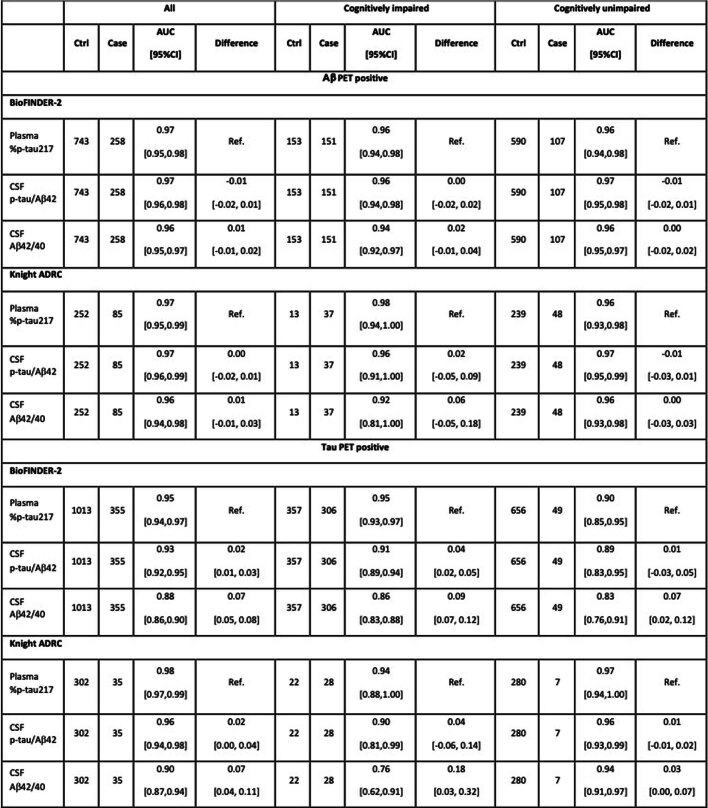
Concordance of fluid and imaging biomarkers of Aβ and tau pathologies

Concordance of fluid biomarkers with Aβ and tau PET positivity in BioFINDER-2 and Knight ADRC participants. AUCs for all, cognitively impaired and cognitively unimpaired groups are shown from left to right. The number of negative (controls) and positive (cases) imaging-based groups are shown in each case. Differences between the statistics using plasma %p-tau217 (reference) and CSF biomarkers are shown together with the mean values. We considered plasma and CSF biomarkers clinically equivalent if the 95% CI of the mean difference included zero and clinically superior if it did not include zero and favored plasma (>0). Aβ PET positivity was assessed as Centiloids ≥ 37. Tau PET positivity was assessed using in-house previously validated cutoffs (SUVR > 1.32 for both cohorts in Braak I–IV). Ctrl, control.

## Data Availability

Pseudonymized data from the BioFINDER-2 study will be shared upon request from a qualified academic investigator for the sole purpose of replicating procedures and results presented in this article and as long as the data transfer is in agreement with European Union legislation on general data protection regulations and decisions by the Swedish Ethical Review Authority and Region Skåne, which should be regulated in a material transfer agreement. Knight ADRC data are available to qualified investigators who have a proposal approved by an institutional committee that meets monthly (https://knightadrc.wustl.edu/Research/ResourceRequest.htm). The study must be approved by an institutional review board to ensure ethical research practices, and investigators must agree to the terms and conditions of the data use agreement, which includes not distributing the data without permission.
