# Supplementary material for: Highly accurate blood test for Alzheimer’s disease is similar or superior to clinical cerebrospinal fluid tests
Source: Nat Med. 2024 Feb 21;30(4):1085–95. doi: 10.1038/s41591-024-02869-z (PMC11031399; doi:10.1038/s41591-024-02869-z)
Supplement: Supplementary file 1 — Supplementary Results: Supplementary Table 1: Comparison among fluid biomarkers on predicting Aβ PET visual read positivity in cognitively impaired patients with in-bag estimates. Supplementary Table 2: Comparison among fluid biomarkers on predicting Aβ positivity in cognitively impaired Knight ADRC individuals with in-bag estimates. Supplementary Fig. 1: Comparison among fluid biomarkers on predicting Aβ PET positivity in cognitively impaired patients of the Knight ADRC cohort with in-bag estimates. Supplementary Fig. 2: Comparison among fluid biomarkers on predicting tau PET positivity in cognitively impaired patients of the Knight ADRC cohort with in-bag estimates. Supplementary Table 3: Comparison among fluid biomarkers on predicting tau PET positivity in cognitively impaired patients with in-bag estimates. Supplementary Table 4: Diagnosis of cognitively impaired participants. Supplementary Table 5: Concordance of fluid biomarkers and AD diagnosis. Supplementary Table 6: Accuracy of plasma %p-tau217 in classifying diagnosis as AD or non-AD in cognitively impaired patients of the BioFINDER-2 cohort. Supplementary Table 7: Comparison among fluid biomarkers on predicting Aβ and tau PET positivity in cognitively impaired patients using a single-cutoff approach with out-of-bag estimates. Supplementary Table 8: Comparison among fluid biomarkers on predicting Aβ and tau PET positivity in cognitively impaired patients using a two-cutoffs approach with out-of-bag estimates. Supplementary Fig. 3: Comparison among fluid biomarkers on predicting Aβ PET positivity in cognitively impaired patients of the BioFINDER-2 cohort with out-of-bag statistics with the bootstrap approach. Supplementary Fig. 4: Comparison among fluid biomarkers on predicting tau PET positivity in cognitively impaired patients of the BioFINDER-2 cohort with out-of-bag statistics with the bootstrap approach. Supplementary Fig. 5: Comparison between plasma p-tau217 and %p-tau217 on predicting Aβ PET positiv [file 41591_2024_2869_MOESM1_ESM.pdf]

nature medicine

Article https://doi.org/10.1038/s41591-024-02869-z

Highly accurate blood test for Alzheimer's disease is similar or superior to clinical cerebrospinal fluid tests

In the format provided by the authors and unedited

Supplementary Results:

Supplementary Table 1: Comparison among fluid biomarkers on predicting Aβ PET visual read positivity in cognitively impaired patients with in-bag estimates

Supplementary Table 2: Comparison among fluid biomarkers on predicting Aβ positivity in cognitively impaired Knight ADRC individuals with in-bag estimates

Supplementary Figure 1: Comparison among fluid biomarkers on predicting Aβ PET positivity in cognitively impaired patients of the Knight ADRC cohort with in-bag estimates

Supplementary Figure 2: Comparison among fluid biomarkers on predicting tau PET positivity in cognitively impaired patients of the Knight ADRC cohort with in-bag estimates

Supplementary Table 3: Comparison among fluid biomarkers on predicting tau PET positivity in cognitively impaired patients with in-bag estimates

Supplementary Table 4: Diagnosis of cognitively impaired participants

Supplementary Table 5: Concordance of fluid biomarkers and AD diagnosis

Supplementary Table 6: Accuracy of plasma %p-tau217 in classifying diagnosis as AD or non-AD in cognitively impaired patients of the BioFINDER-2 cohort

Supplementary Table 7: Comparison among fluid biomarkers on predicting Aβ and tau PET positivity in cognitively impaired patients using a single cut-off approach with out-of-bag estimates

Supplementary Table 8: Comparison among fluid biomarkers on predicting Aβ- and tau PET positivity in cognitively impaired patients using a two cut-offs approach with out-of-bag estimates

Supplementary Figure 3: Comparison among fluid biomarkers on predicting Aβ PET positivity in cognitively impaired patients of the BioFINDER-2 cohort with out-of-bag statistics with the bootstrap approach

Supplementary Figure 4: Comparison among fluid biomarkers on predicting tau PET positivity in cognitively impaired patients of the BioFINDER-2 cohort with out-of-bag statistics with the bootstrap approach

Supplementary Figure 5: Comparison between plasma p-tau217 and %p-tau217 on predicting Aβ PET positivity in cognitively impaired patients of the BioFINDER-2 cohort with in-bag estimates

Supplementary Figure 6: Comparison between plasma p-tau217 and %p-tau217 on predicting tau PET positivity in cognitively impaired patients of the BioFINDER-2 cohort with in-bag estimates

Supplementary Table 9: Comparison between plasma p-tau217 and %p-tau217 on predicting Aβ and tau PET positivity in cognitively impaired patients using a single cut-off approach with in-bag estimates

Supplementary Table 10: Comparison between plasma p-tau217 and %p-tau217 on predicting Aβ and tau PET positivity in cognitively impaired patients using a two cut-offs approach with in-bag estimates

Supplementary Figure 7: Longitudinal trajectories of plasma %p-tau217 in Knight ADRC participants

Supplementary Methods:

Plasma %p-tau217 analysis by IP-MS

Comparison of plasma %p-tau217 measurements in Knight ADRC and BioFINDER-2

UCSF cohort

References

Single cut-off approach								
	Accuracy		PPV		NPV		Sensitivity	
	Mean	Difference	Mean	Difference	Mean	Difference	Mean	Difference
**Aβ PET Visual read positive**								
Plasma %p-tau217	0.91 [0.85,0.94]	Ref.	0.92 [0.89,0.94]	Ref.	0.91 [0.79,0.97]	Ref.	0.91 [0.78,0.97]	Ref.
CSF p-tau/Aβ42	0.93 [0.90,0.95]	-0.01 [-0.08, 0.03]	0.92 [0.90,0.94]	0.00 [-0.03, 0.02]	0.93 [0.88,0.98]	-0.03 [-0.13, 0.05]	0.94 [0.88,0.98]	-0.03 [-0.15, 0.05]
CSF Aβ42/40	0.90 [0.83,0.94]	0.02 [-0.05, 0.09]	0.91 [0.89,0.94]	0.00 [-0.02, 0.03]	0.88 [0.76,0.97]	0.03 [-0.09, 0.15]	0.88 [0.75,0.97]	0.03 [-0.10, 0.17]
**Two cut-off approach**								
	Accuracy		PPV		NPV		Number of Intermediate participants	
	Mean	Difference	Mean	Difference	Mean	Difference	Mean	Difference*
Plasma %p-tau217	0.95 [0.94,0.96]	Ref.	0.95 [0.94,0.97]	Ref.	0.95 [0.94,0.97]	Ref.	15.2 [3,0,25.3]	Ref.
CSF p-tau/Aβ42	0.95 [0.94,0.96]	0.00 [-0.01, 0.01]	0.96 [0.93,0.97]	0.00 [-0.02, 0.02]	0.95 [0.94,0.97]	0.00 [-0.02, 0.02]	10.0 [0.0,29.3]	-0.05 [-0.17, 0.12]
CSF Aβ42/40	0.95 [0.93,0.96]	0.00 [-0.01, 0.02]	0.94 [0.89,0.96]	0.01 [-0.01, 0.05]	0.95 [0.94,0.97]	0.00 [-0.02, 0.02]	18.5 [6.6,38.5]	0.03 [-0.12, 0.24]

Supplementary Table 1: Comparison among fluid biomarkers on predicting Aβ PET visual read positivity in cognitively impaired patients with in-bag estimates

Comparison of accuracy, PPV, NPV and sensitivity among fluid biomarkers on predicting Aβ PET visual read positivity in cognitively impaired patients. For the single cut-off approach, the cut-off of fluid biomarkers was derived by maximizing sensitivity fixing specificity at 90% against each imaging outcome. For the two cut-offs approach, the lower cut-off was obtained by maximizing specificity with sensitivity fixed at 95%, whereas the upper cut-off was obtained by maximizing sensitivity fixing specificity at 95%.

Participants that fall between these two cut-offs were classified in the intermediate group. Differences between the statistics using plasma %p-tau217 (reference) and CSF biomarkers are shown together with the mean values. We considered plasma and CSF biomarkers clinically equivalent if the 95%CI of the mean difference included zero and clinically superior if it did not include zero and favored plasma (>0). Values in all cells represent mean[95%CI] of bootstrapped sample. *Differences in number of participants in the intermediate group have been scaled to a maximum of one to be comparable to the other differences. Aβ PET positivity was assessed following FDA-approved protocol by an experienced reader (LEC).

Abbreviations: Aβ, amyloid-β; CI, confidence interval; CSF, cerebrospinal fluid; NPV, negative predictive value; PPV, positive predictive value.

Single cut-off approach								
	Accuracy		PPV		NPV		Sensitivity	
	Mean	Difference	Mean	Difference	Mean	Difference	Mean	Difference
Plasma %p-tau217	0.94 [0.72,1.00]	Ref.	0.99 [0.97,1.00]	Ref.	0.89 [0.48,1.00]	Ref.	0.93 [0.62,1.00]	Ref.
CSF p-tau/Aβ42	0.91 [0.68,1.00]	0.03 [-0.24, 0.30]	0.99 [0.97,1.00]	0.00 [-0.03, 0.03]	0.82 [0.45,1.00]	0.07 [-0.46, 0.54]	0.89 [0.57,1.00]	0.04 [-0.32, 0.41]
CSF Aβ42/40	0.78 [0.44,0.98]	0.16 [-0.20, 0.56]	0.98 [0.96,1.00]	0.01 [-0.03, 0.03]	0.62 [0.32,1.00]	0.26 [-0.36, 0.68]	0.72 [0.24,1.00]	0.21 [-0.27, 0.76]
Two cut-off approach								
	Accuracy		PPV		NPV		Number of Intermediate participants	
	Mean	Difference	Mean	Difference	Mean	Difference	Mean	Difference*
Plasma %p-tau217	0.99 [0.97,1.00]	Ref.	1.00 [1.00,1.00]	Ref.	0.97 [0.91,1.00]	Ref.	14.2 [0.0,32.0]	Ref.
CSF p-tau/Aβ42	0.99 [0.97,1.00]	0.00 [-0.03, 0.03]	1.00 [1.00,1.00]	0.00 [0.00, 0.00]	0.96 [0.91,1.00]	0.01 [-0.08, 0.09]	20.2 [0.0,48.0]	0.06 [-0.28, 0.42]
CSF Aβ42/40	0.98 [0.94,1.00]	0.01 [-0.03, 0.05]	1.00 [1.00,1.00]	0.00 [0.00, 0.00]	0.94 [0.86,1.00]	0.03 [-0.08, 0.12]	42.8 [4.0,72.0]	0.29 [-0.18, 0.64]

Supplementary Table 2: Comparison among fluid biomarkers on predicting Aβ positivity in cognitively impaired Knight ADRC individuals with in-bag estimates

Comparison estimates among fluid biomarkers on predicting Aβ PET positivity in cognitively impaired individuals. For the single cut-off approach, fluid biomarkers' cut-offs were derived by maximizing sensitivity fixing specificity at 90% against each imaging outcome. For the two cut-offs approach, the lower cut-off was obtained by maximizing specificity with sensitivity fixed at 95%, whereas the upper cut-off was obtained by maximizing sensitivity fixing specificity at 95%. Participants that fall between these two

cut-offs were classified in the intermediate group. Differences between the statistics using plasma %p-tau217 (reference) and CSF biomarkers are shown together with the mean values. We considered plasma and CSF biomarkers clinically equivalent if the 95%CI of the mean difference included zero and clinically superior if it did not include zero and favored plasma (>0). We considered plasma and CSF biomarkers clinically equivalent if the 95%CI of the mean difference included zero. *Differences in number of participants in the intermediate group have been scaled to a maximum of one to be comparable to the other differences. Aβ PET positivity was assessed as Centiloids≥37.

Abbreviations: Aβ, amyloid-β; CI, confidence interval; CSF, cerebrospinal fluid; NPV, negative predictive value; PPV, positive predictive value.

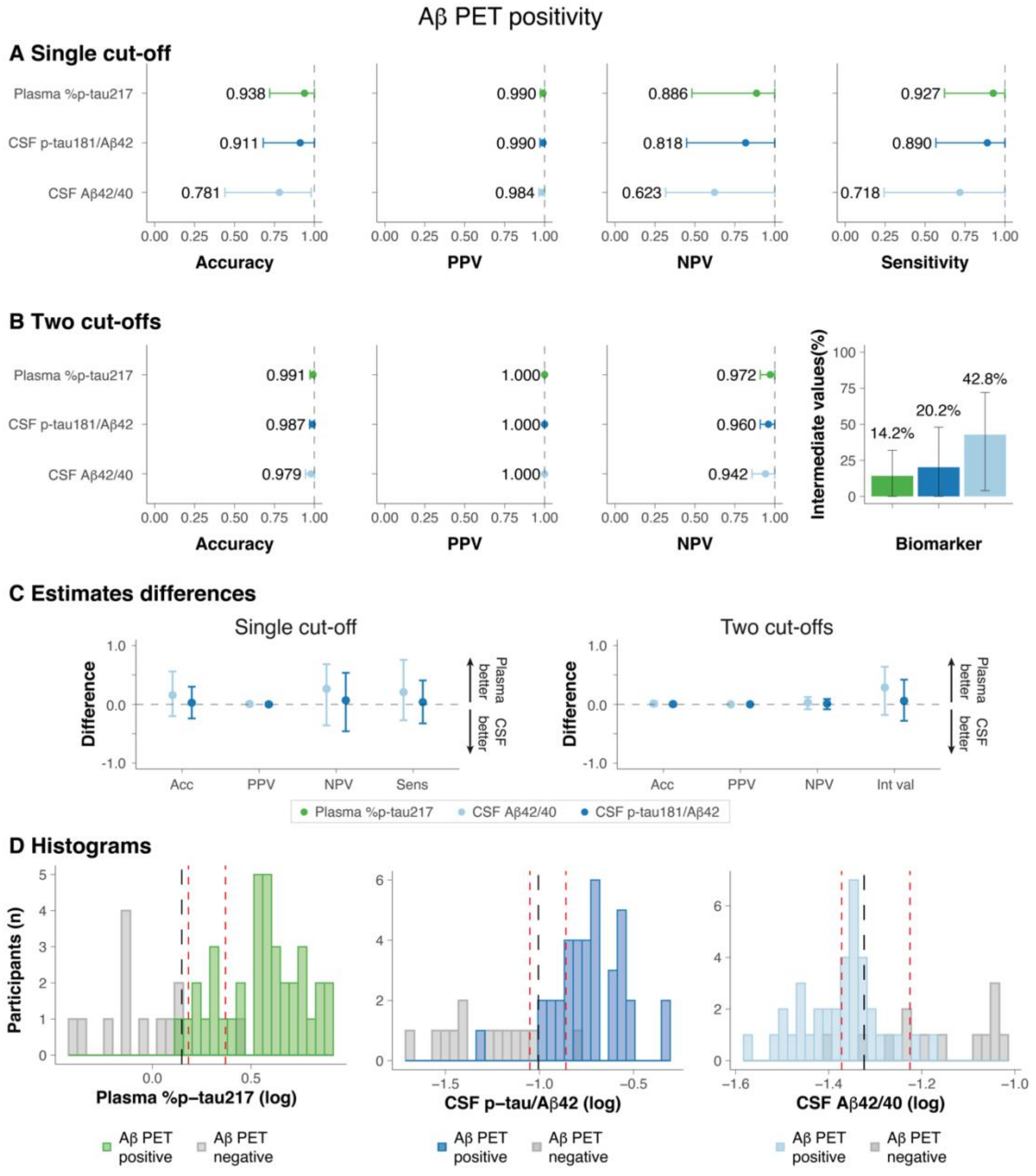

Supplementary Figure 1: Comparison among fluid biomarkers on predicting Aβ PET positivity in cognitively impaired patients of the Knight ADRC cohort with in-bag estimates

Prediction of Aβ PET positivity in cognitively impaired participants from the Knight ADRC (n=50), using a one cut-off (A) and two cut-offs (B) approach, respectively. In the first approach, the cut-off was calculated maximizing sensitivity fixing specificity at 90%. In the second approach, the lower cut-off was obtained by maximizing specificity with sensitivity fixed at 95%, whereas the upper cut-off was

obtained by maximizing sensitivity fixing specificity at 95%. Participants that fall between these two cut-offs were classified in the intermediate group. Dots and error bars represent the actual statistic and 95%CI (from bootstrapped n=1,000 samples with replacement), respectively. Bootstrapped differences (n=1,000 resamples with replacement stratifying by the output) between the statistics using plasma %p-tau217 (reference) and CSF biomarkers are shown in C for both single and two cut-offs. A horizontal dashed line is plotted at zero representing the lack of difference between plasma and CSF biomarkers. We considered plasma and CSF biomarkers clinically equivalent if the 95%CI of the mean difference included zero and clinically superior if it did not include zero and favored plasma (>0). Dots and error bars represent the mean and 95%CI estimate from a bootstrapped sample. Vertical dashed lines represent the maximal statistical value possible (1). For the intermediate values plots, coloured bars represent the actual percentage and error bar the 95%CI. Histograms (D) represent the distribution of the data coloured by the imaging biomarker status. Vertical black line represents the cut-off derived from the first approach (A), and red lines represent the lower and upper cut-offs from the second approach (B). Aβ PET positivity was assessed as Centiloids≥37.

Abbreviations: Aβ, amyloid-β; CI, confidence interval; CSF, cerebrospinal fluid; NPV, negative predictive value; PPV, positive predictive value.

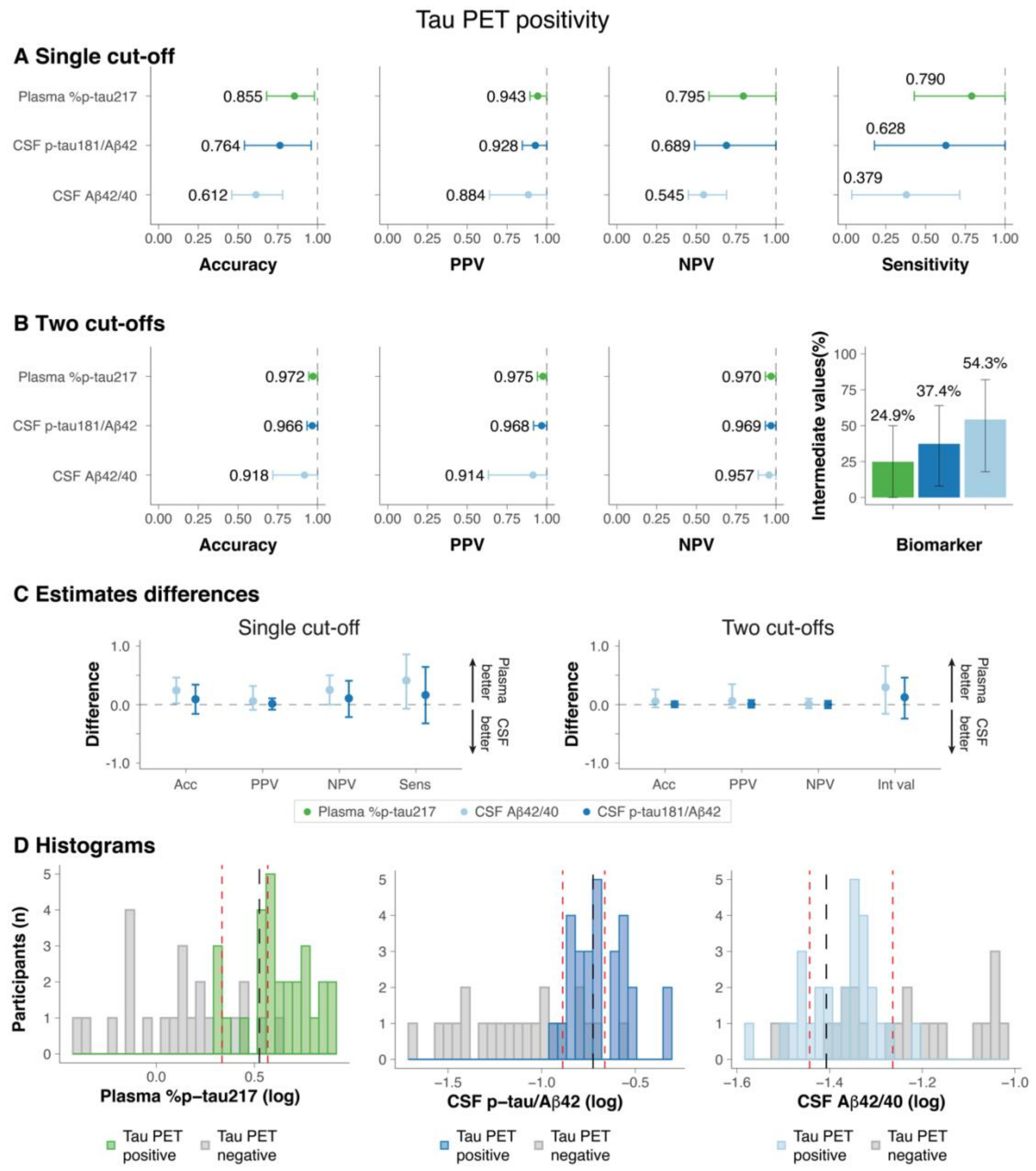

Supplementary Figure 2: Comparison among fluid biomarkers on predicting tau PET positivity in cognitively impaired patients of the Knight ADRC cohort with in-bag estimates

Prediction of tau PET positivity in cognitively impaired participants from the Knight ADRC (n=50), using a one cut-off (A) and two cut-offs (B) approach, respectively. In the first approach, the cut-off was calculated maximizing sensitivity fixing specificity at 90%. In the second approach, the lower cut-off was obtained by maximizing specificity with sensitivity fixed at 95%, whereas the upper cut-off was obtained by maximizing sensitivity fixing specificity at 95%. Participants that fall between these two cut-offs were classified in the intermediate group. Dots and

error bars represent the actual statistic and 95%CI (from bootstrapped n=1,000 samples with replacement), respectively. Bootstrapped differences (n=1,000 resamples with replacement stratifying by the output) between the statistics using plasma %p-tau217 (reference) and CSF biomarkers are shown in C for both single and two cut-offs. A horizontal dashed line is plotted at zero representing the lack of difference between plasma and CSF biomarkers. We considered plasma and CSF biomarkers clinically equivalent if the 95%CI of the mean difference included zero and clinically superior if it did not include zero and favored plasma (>0). Differences in number of participants in the intermediate group have been scaled to a maximum of one to be comparable to the other differences. Dots and error bars represent the mean and 95%CI estimate from a bootstrapped sample. Vertical dashed lines represent the maximal statistical value possible (1). For the intermediate values plots, coloured bars represent the actual percentage and error bar the 95%CI. Histograms (D) represent the distribution of the data coloured by the imaging biomarker status. Vertical black line represents the cut-off derived from the first approach (A), and red lines represent the lower and upper cut-offs from the second approach (B). Tau PET positivity was assessed using an in-house previously validated cut-off (SUVR>1.32).

Abbreviations: Aβ, amyloid-β; CI, confidence interval; CSF, cerebrospinal fluid; NPV, negative predictive value; PPV, positive predictive value.

Single cut-off approach								
	Accuracy		PPV		NPV		Sensitivity	
	Mean	Difference	Mean	Difference	Mean	Difference	Mean	Difference
Plasma %p-tau217	0.85 [0.68,0.98]	Ref.	0.94 [0.89,1.00]	Ref.	0.80 [0.58,1.00]	Ref.	0.79 [0.43,1.00]	Ref.
CSF p-tau/Aβ42	0.76 [0.54,0.96]	0.09 [-0.16, 0.34]	0.93 [0.85,1.00]	0.01 [-0.09, 0.11]	0.69 [0.49,1.00]	0.11 [-0.21, 0.41]	0.63 [0.18,1.00]	0.16 [-0.32, 0.64]
CSF Aβ42/40	0.61 [0.46,0.78]	0.24 [0.02, 0.46]	0.88 [0.64,1.00]	0.06 [-0.09, 0.32]	0.55 [0.45,0.69]	0.25 [0.00, 0.50]	0.38 [0.04,0.71]	0.41 [-0.07, 0.86]
Two cut-off approach								
	Accuracy		PPV		NPV		Number of Intermediate participants	
	Mean	Difference	Mean	Difference	Mean	Difference	Mean	Difference*
Plasma %p-tau217	0.97 [0.94,1.00]	Ref.	0.98 [0.94,1.00]	Ref.	0.97 [0.93,1.00]	Ref.	24.9 [0.0,50.0]	Ref.
CSF p-tau/Aβ42	0.97 [0.93,1.00]	0.01 [-0.05, 0.06]	0.97 [0.92,1.00]	0.01 [-0.05, 0.08]	0.97 [0.93,1.00]	0.00 [-0.06, 0.06]	37.4 [8.0,64.0]	0.12 [-0.24, 0.46]
CSF Aβ42/40	0.92 [0.72,1.00]	0.05 [-0.05, 0.26]	0.91 [0.63,1.00]	0.06 [-0.05, 0.35]	0.96 [0.89,1.00]	0.01 [-0.06, 0.10]	54.3 [18.0,82.0]	0.29 [-0.16, 0.66]

Supplementary Table 3: Comparison among fluid biomarkers on predicting tau PET positivity in cognitively impaired patients in Knight ADRC with in-bag estimates

Comparison estimates among fluid biomarkers on predicting tau PET positivity in cognitively impaired individuals from the Knight ADRC For the single cut-off approach, fluid biomarkers' cut-offs were derived by maximizing sensitivity fixing specificity at 90% against each imaging outcome. For the two cut-offs approach, the lower cut-off was obtained by maximizing specificity with sensitivity fixed at 95%, whereas the upper cut-off was obtained by maximizing sensitivity fixing specificity at 95%. Participants that

fall between these two cut-offs were classified in the intermediate group. Differences between the statistics using plasma %p-tau217 (reference) and CSF biomarkers are shown together with the mean values. We considered plasma and CSF biomarkers clinically equivalent if the 95%CI of the mean difference included zero and clinically superior if it did not include zero and favored plasma (>0). *Differences in number of participants in the intermediate group have been scaled to a maximum of one to be comparable to the other differences. Tau PET positivity was assessed using an in-house previously validated cut-off (SUVR>1.32 for both cohorts). Abbreviations: Aβ, amyloid-β; CI, confidence interval; CSF, cerebrospinal fluid; NPV, negative predictive value; PPV, positive predictive value.

Diagnosis	N (%)
Alzheimer's disease with dementia	212 (30.2%)
Mild cognitive impairment due to Alzheimer's disease	134 (19.1%)
Mild cognitive impairment not due to Alzheimer's disease	102 (14.5%)
Parkinsonian disorders and atypical parkinsonism diseases (Parkinson's disease, progressive supranuclear palsy, corticobasal syndrome, multiple system atrophy)	79 (11.3%)
Dementia with Lewy bodies	47 (6.7%)
Vascular dementia	41 (5.8%)
Frontotemporal disease	36 (5.1%)
Neurodegenerative disorder, not otherwise specified	28 (4.0%)
Primary progressive aphasia	11 (1.6%)
Non-neurodegenerative	8 (1.1%)
Normal pressure hydrocephalus	4 (0.6%)

Supplementary Table 4: Diagnosis of cognitively impaired participants

Number (%) of participants of each diagnosis in the cognitively impaired group of the BioFINDER-2 cohort.

	Controls	Cases	AUC[95%CI]	Difference AUC
**AD diagnosis**				
**BioFINDER-2**				
Plasma %p-tau217	356	346	0.94[0.92,0.96]	Ref.
CSF p-tau/Aβ42	356	346	0.95[0.93,0.96]	-0.01[-0.02, 0.01]
CSF Aβ42/40	356	346	0.93[0.91,0.95]	0.02[-0.01, 0.04]

Supplementary Table 5: Concordance of fluid biomarkers and AD diagnosis

Concordance of fluid biomarkers with AD diagnosis in cognitively impaired participants of the BioFINDER-2 cohort. Number of non-AD (controls) and AD (cases) diagnosis are shown. Differences between the statistics using plasma %p-tau217 (reference) and CSF biomarkers are shown together with the mean values. We considered plasma and CSF biomarkers clinically equivalent if the 95%CI of the mean difference included zero and clinically superior if it did not

include zero and favored plasma (>0). Differences in AUCs were calculated using the DeLong's test. AD diagnosis was assessed based on the Diagnostic and Statistical Manual of Mental Disorders [Fifth Edition] AD criteria, and was also supported by CSF biomarkers for clinical practice and longitudinal clinical diagnosis in many cases.

Abbreviations: Aβ, amyloid-β; AD, Alzheimer's disease; AUC, area under the curve; CI, confidence interval; CSF, cerebrospinal fluid.

**AD diagnosis**				
**Single cut-off approach**				
	**Accuracy[95%CI]**	**PPV[95%CI]**	**NPV[95%CI]**	**Sensitivity[95%CI]**
Plasma %p-tau217	0.86[0.82,0.89]	0.89[0.87,0.91]	0.84[0.77,0.89]	0.82[0.72,0.88]
**Two cut-off approach**				
	**Accuracy[95%CI]**	**PPV[95%CI]**	**NPV[95%CI]**	**Percentage intermediate[95%CI]**
Plasma %p-tau217	0.94[0.93,0.95]	0.93[0.91,0.95]	0.94[0.94,0.95]	24.2[16.8,32.5]

Supplementary Table 6: Accuracy of plasma %p-tau217 in classifying diagnosis as AD or non-AD in cognitively impaired patients of the BioFINDER-2 cohort

Mean and 95%CI estimates using a single cut-off or two cut-offs. For the single cut-off, we calculated the accuracy, PPV, NPV and sensitivity of the plasma %p-tau217 biomarker on predicting AD diagnosis in cognitively impaired patients. The cut-off was derived by maximizing sensitivity while fixing specificity at 90% against AD diagnosis. For the second approach, we calculated accuracy, PPV, NPV and number participants categorised in the intermediate group. The lower cut-off was obtained by maximizing specificity with sensitivity fixed at 95%, whereas the upper cut-off was obtained by maximizing sensitivity while fixing specificity at 95%. Participants that fall between these two cut-offs were classified in the intermediate group. AD diagnosis was assessed based on the Diagnostic and Statistical Manual of Mental Disorders [Fifth Edition] AD criteria, and was also supported by CSF biomarkers for clinical practice and longitudinal clinical diagnosis in many cases.

Abbreviations: Aβ, amyloid-β; AD, Alzheimer's disease; CI, confidence interval; CSF, cerebrospinal fluid; NPV, negative predictive value; PPV, positive predictive value.

	Accuracy		PPV		NPV		Sensitivity	
	**Mean**	**Difference**	**Mean**	**Difference**	**Mean**	**Difference**	**Mean**	**Difference**
**Aβ PET positive**								
**Bootstrap BioFINDER-2 cut-offs**								
Plasma %p-tau217	0.89[0.85, 0.93]	Ref.	0.90[0.81, 0.98]	Ref.	0.89[0.82, 0.96]	Ref.	0.89[0.78, 0.97]	Ref.
CSF p-tau/Aβ42	0.90[0.85, 0.94]	-0.01 [-0.07, 0.04]	0.90[0.82, 0.98]	0.00 [-0.09, 0.09]	0.90[0.81, 0.98]	-0.01 [-0.11, 0.09]	0.90[0.78, 0.98]	-0.01 [-0.14, 0.12]
CSF Aβ42/40	0.86[0.79, 0.91]	0.03 [-0.03, 0.11]	0.89[0.81, 0.97]	0.00 [-0.10, 0.10]	0.85[0.71, 0.96]	0.04 [-0.09, 0.18]	0.83[0.59, 0.96]	0.06 [-0.11, 0.27]
**External cut-offs**								
Plasma %p-tau217	0.91[0.88,0.94]	Ref.	0.89[0.85,0.93]	Ref.	0.93[0.89,0.97]	Ref.	0.93[0.89,0.97]	Ref.
CSF p-tau/Aβ42	0.89[0.86,0.92]	0.02 [-0.02, 0.06]	0.91[0.87,0.95]	-0.02 [-0.07, 0.03]	0.88[0.83,0.92]	0.06 [0.00, 0.11]	0.87[0.81,0.92]	0.07 [0.01, 0.13]
CSF Aβ42/40	0.80[0.76,0.85]	0.11 [0.06, 0.15]	0.91[0.86,0.96]	-0.02 [-0.08, 0.04]	0.74[0.69,0.78]	0.19[0.14, 0.24]	0.67[0.59,0.74]	0.26 [0.19, 0.34]
**Tau PET positive**								
**Bootstrap BioFINDER-2 cut-offs**								
Plasma %p-tau217	0.88[0.85, 0.91]	Ref.	0.88[0.81, 0.94]	Ref.	0.88[0.82, 0.93]	Ref.	0.85[0.75, 0.93]	Ref.
CSF p-tau/Aβ42	0.81[0.77, 0.85]	0.06 [0.02, 0.10]	0.86[0.80, 0.93]	0.02 [-0.06, 0.09]	0.79[0.73, 0.86]	0.09 [0.01, 0.16]	0.71[0.58, 0.84]	0.14 [0.00, 0.26]
CSF Aβ42/40	0.67[0.64, 0.72]	0.20 [0.15, 0.25]	0.78[0.71, 0.88]	0.10 [-0.01, 0.18]	0.64[0.60, 0.70]	0.23 [0.17, 0.30]	0.41[0.29, 0.57]	0.44 [0.28, 0.58]

Supplementary Table 7: Comparison among fluid biomarkers on predicting Aβ and tau PET positivity in cognitively impaired patients using a single cut-off approach with out-of-bag estimates

Comparison of accuracy, PPV, NPV and sensitivity among fluid biomarkers on predicting Aβ- and tau PET positivity in cognitively impaired patients. The cut-off of fluid biomarkers was derived by maximizing sensitivity fixing specificity at 90% against each

imaging outcome. The bootstrap approach derives the cut-off in a bootstrapped sample of the same cohort (same sample size with resampling) and calculates the statistics in the remaining participants of the same sample not included in the cut-off derivation. The external cut-off method derives the cut-offs in independent cohorts. Plasma %p-tau217 cut-offs were derived in the Knight ADRC cohort, and CSF biomarkers were derived in the UCSF cohort. Differences between the statistics using plasma %p-tau217 (reference) and CSF biomarkers are shown together with the mean values. We considered plasma and CSF biomarkers clinically equivalent if the 95%CI of the mean difference included zero and clinically superior if it did not include zero and favored plasma (>0). Aβ PET positivity was assessed as Centiloids≥37. Tau PET positivity was assessed using in-house previously validated cut-off (SUVR>1.32).

Abbreviations: Aβ, amyloid-β; CI, confidence interval; CSF, cerebrospinal fluid; NPV, negative predictive value; PPV, positive predictive value.

	Accuracy		PPV		NPV		Number of Intermediate participants	
	Mean	Difference	Mean	Difference	Mean	Difference	Mean	Difference*
**Aβ PET positive**								
**Bootstrap BioFINDER-2 cut-offs**								
Plasma %p-tau217	0.94 [0.87, 0.99]	Ref.	0.94 [0.85, 1.00]	Ref.	0.94 [0.86, 1.00]	Ref.	15.2 [3.6, 27.2]	Ref.
CSF p-tau/Aβ42	0.94 [0.88, 0.99]	0.00 [-0.07, 0.07]	0.94 [0.85, 1.00]	0.01 [-0.08, 0.08]	0.95 [0.86, 1.00]	0.00 [-0.10, 0.09]	15.9 [3.2, 34.3]	0.01 [-0.16, 0.22]
CSF Aβ42/40	0.93 [0.87, 0.99]	0.01 [-0.07, 0.08]	0.92 [0.84, 1.00]	0.02 [-0.09, 0.11]	0.94 [0.86, 1.00]	0.00 [-0.10, 0.09]	24.9 [8.5, 41.9]	0.10 [-0.10, 0.29]
**External cut-offs**								
Plasma %p-tau217	0.93 [0.90,0.96]	Ref.	0.99 [0.96,1.00]	Ref.	0.90 [0.86,0.95]	Ref.	25.0 [20.4,29.6]	Ref.
CSF p-tau/Aβ42	0.89 [0.85,0.92]	0.04 [0.00, 0.09]	0.94 [0.89,0.98]	0.05 [0.01, 0.10]	0.86 [0.82,0.91]	0.04 [-0.02, 0.10]	15.1 [11.0,18.9]	-0.10 [-0.16, -0.05]
CSF Aβ42/40	0.81 [0.77,0.86]	0.12 [0.06, 0.17]	0.92 [0.86,0.98]	0.06 [0.00, 0.13]	0.77 [0.73,0.82]	0.13 [0.06, 0.19]	18.4 [14.5,22.4]	-0.07 [-0.13, 0.00]
**Tau PET positive**								
**Bootstrap BioFINDER-2 cut-offs**								
Plasma %p-tau217	0.94 [0.89, 0.97]	Ref.	0.93 [0.87, 0.98]	Ref.	0.94 [0.89, 0.99]	Ref.	19.3 [10.8, 28.0]	Ref.
CSF p-tau/Aβ42	0.92 [0.87, 0.97]	0.01 [-0.03, 0.06]	0.89 [0.83, 0.96]	0.03 [-0.05, 0.12]	0.94 [0.88, 0.99]	0.00 [-0.04, 0.05]	33.9 [23.1, 44.1]	0.15 [0.02, 0.26]
CSF Aβ42/40	0.90 [0.84, 0.95]	0.04 [-0.02, 0.09]	0.81 [0.70, 0.92]	0.12 [0.00, 0.24]	0.94 [0.87, 0.99]	0.01 [-0.05, 0.07]	48.7 [39.3, 58.9]	0.29 [0.18, 0.41]

Supplementary Table 8: Comparison among fluid biomarkers on predicting Aβ and tau PET positivity in cognitively impaired patients using a two cut-offs approach with out-of-bag estimates

Comparison of accuracy, PPV, NPV and number participants categorised in the intermediate group among fluid biomarkers on predicting Aβ- and tau PET positivity in cognitively impaired patients. The lower cut-off was obtained by maximizing specificity with sensitivity fixed at 95%, whereas the upper cut-off was obtained by maximizing sensitivity fixing specificity at 95%. Participants that fall between these two cut-offs were classified in the intermediate group. The bootstrap approach derives the cut-off in a bootstrapped sample of the same cohort (same sample size with resampling) and calculates the statistics in the remaining participants of the same sample not included in the cut-off derivation. The external cut-off method derives the cut-offs in independent cohorts. Plasma %p-tau217 cut-offs were derived in the Knight ADRC cohort, and CSF biomarkers were derived in the UCSF cohort. Differences between the statistics using plasma %p-tau217 (reference) and CSF biomarkers are shown together with the mean values. Differences in number of participants in the intermediate group has been scaled to a maximum of one to be comparable to the other differences. We considered plasma and CSF biomarkers clinically equivalent if the 95%CI of the mean difference included zero and clinically superior if it did not include zero and favored plasma (>0). *Differences in number of participants in the intermediate group have been scaled to a maximum of one to be comparable to the other differences. Aβ PET positivity was assessed as Centiloids≥37. Tau PET positivity was assessed using in-house previously validated cut-off (SUVR>1.32).

Abbreviations: Aβ, amyloid-β; CI, confidence interval; CSF, cerebrospinal fluid; NPV, negative predictive value; PPV, positive predictive value.

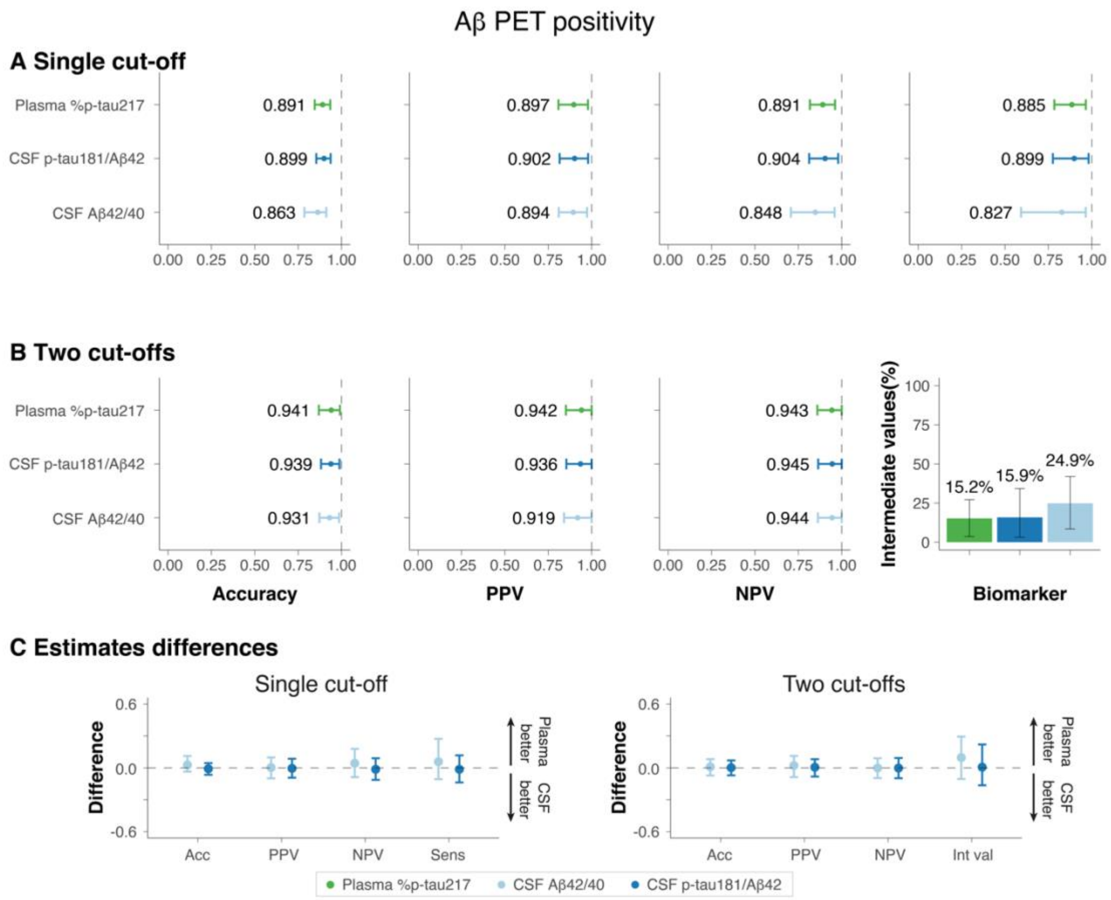

Supplementary Figure 3: Comparison among fluid biomarkers on predicting Aβ PET positivity in cognitively impaired patients of the BioFINDER-2 cohort with out-of-bag statistics with the bootstrap approach

Prediction of Aβ PET positivity in cognitively impaired participants from the BioFINDER-2 cohort, using a single cut-off (A) and two cut-offs (B) approaches, respectively. In the first approach, the cut-off was calculated maximizing sensitivity fixing specificity at 90%. In the second approach, the lower cut-off was obtained by maximizing specificity with sensitivity fixed at 95%, whereas the upper cut-off was obtained by maximizing sensitivity fixing specificity at 95%. Participants that fall between these two cut-offs were classified in the intermediate group. Dots and error bars represent the actual statistic and 95%CI (from bootstrapped n=1,000 samples with replacement), respectively. The bootstrap approach derives the cut-off in a bootstrapped sample of the same cohort (same sample size with resampling) and calculates the statistics in the remaining participants of the same sample not included in the cut-off derivation. Bootstrapped differences (n=1,000 resamples with replacement stratifying by the output) between the statistics using plasma %p-tau217 (reference) and CSF

biomarkers are shown in C for both single and two cut-offs. A horizontal dashed line is plotted at zero representing the lack of difference between plasma and CSF biomarkers. We considered plasma and CSF biomarkers clinically equivalent if the 95%CI of the mean difference included zero and clinically superior if it did not include zero and favored plasma (>0). Differences in number of participants in the intermediate group have been scaled to a maximum of one to be comparable to the other differences. Dots and error bars represent the mean and 95%CI estimate from a bootstrapped sample. Vertical dashed lines represent the maximal statistical value possible (1). For the intermediate values plots, coloured bars represent the actual percentage and error bar the 95%CI. Aβ PET positivity was assessed as Centiloids≥37.

Abbreviations: Aβ, amyloid-β; CI, confidence interval; CSF, cerebrospinal fluid; NPV, negative predictive value; PPV, positive predictive value.

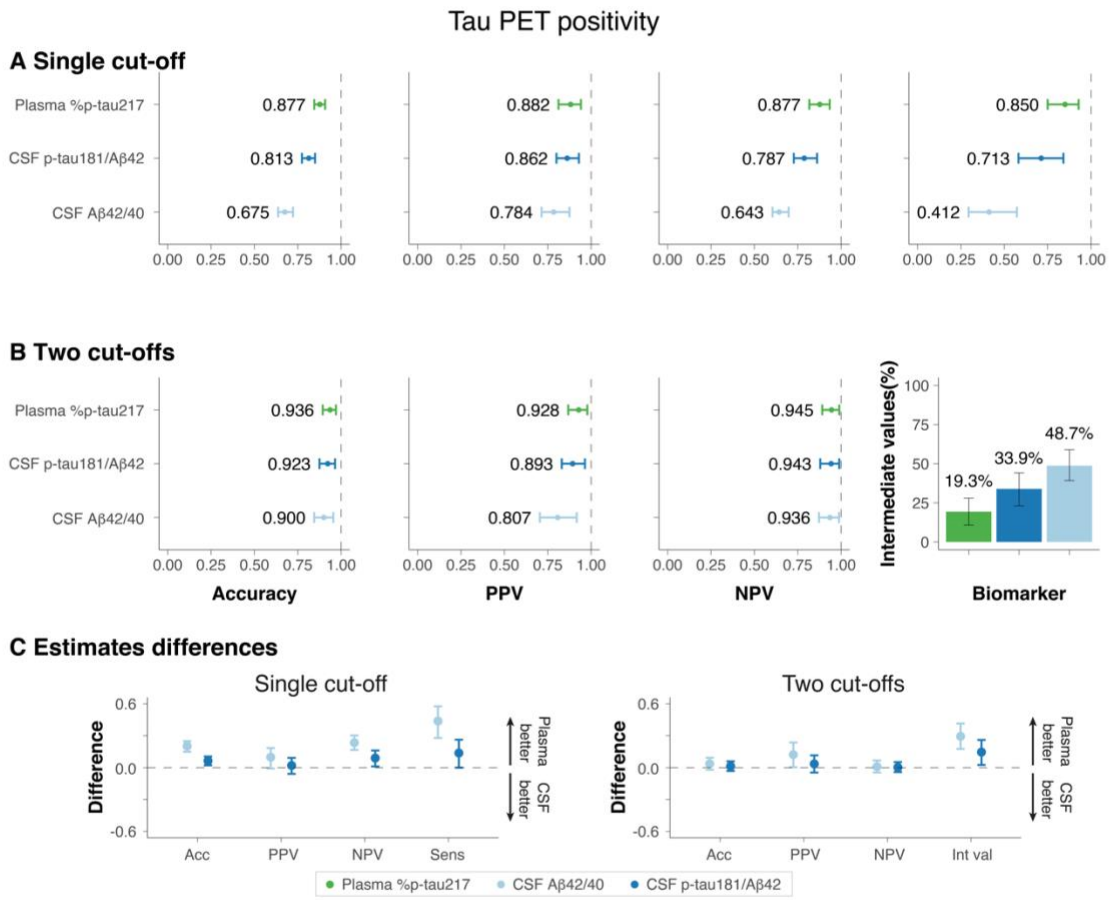

Supplementary Figure 4: Comparison among fluid biomarkers on predicting tau PET positivity in cognitively impaired patients of the BioFINDER-2 cohort with out-of-bag statistics with the bootstrap approach

Prediction of Aβ PET positivity in cognitively impaired participants from the BioFINDER-2 cohort, using a single cut-off (A) and two cut-offs (B) approaches, respectively. In the first approach, the cut-off was calculated maximizing sensitivity fixing specificity at 90%. In the second approach, the lower cut-off was obtained by maximizing specificity with sensitivity fixed at 95%, whereas the upper cut-off was obtained by maximizing sensitivity fixing specificity at 95%. Participants that fall between these two cut-offs were classified in the intermediate group. Dots and error bars represent the actual statistic and 95%CI (from bootstrapped n=1,000 samples with replacement), respectively. The bootstrap approach derives the cut-off in a bootstrapped sample of the same cohort (same sample size with resampling) and calculates the statistics in the remaining participants of the same sample not included in the cut-off derivation. Bootstrapped differences (n=1,000 resamples with replacement stratifying by the output) between the statistics using plasma %p-tau217 (reference) and CSF

biomarkers are shown in C for both single and two cut-offs. A horizontal dashed line is plotted at zero representing the lack of difference between plasma and CSF biomarkers. We considered plasma and CSF biomarkers clinically equivalent if the 95%CI of the mean difference included zero and clinically superior if it did not include zero and favored plasma (>0). Differences in number of participants in the intermediate group have been scaled to a maximum of one to be comparable to the other differences. Dots and error bars represent the mean and 95%CI estimate from a bootstrapped sample. Vertical dashed lines represent the maximal statistical value possible (1). For the intermediate values plots, coloured bars represent the actual percentage and error bar the 95%CI. Tau PET positivity was assessed using an in-house previously validated cut-off (SUVR>1.32).

Abbreviations: Aβ, amyloid-β; CI, confidence interval; CSF, cerebrospinal fluid; NPV, negative predictive value; PPV, positive predictive value.

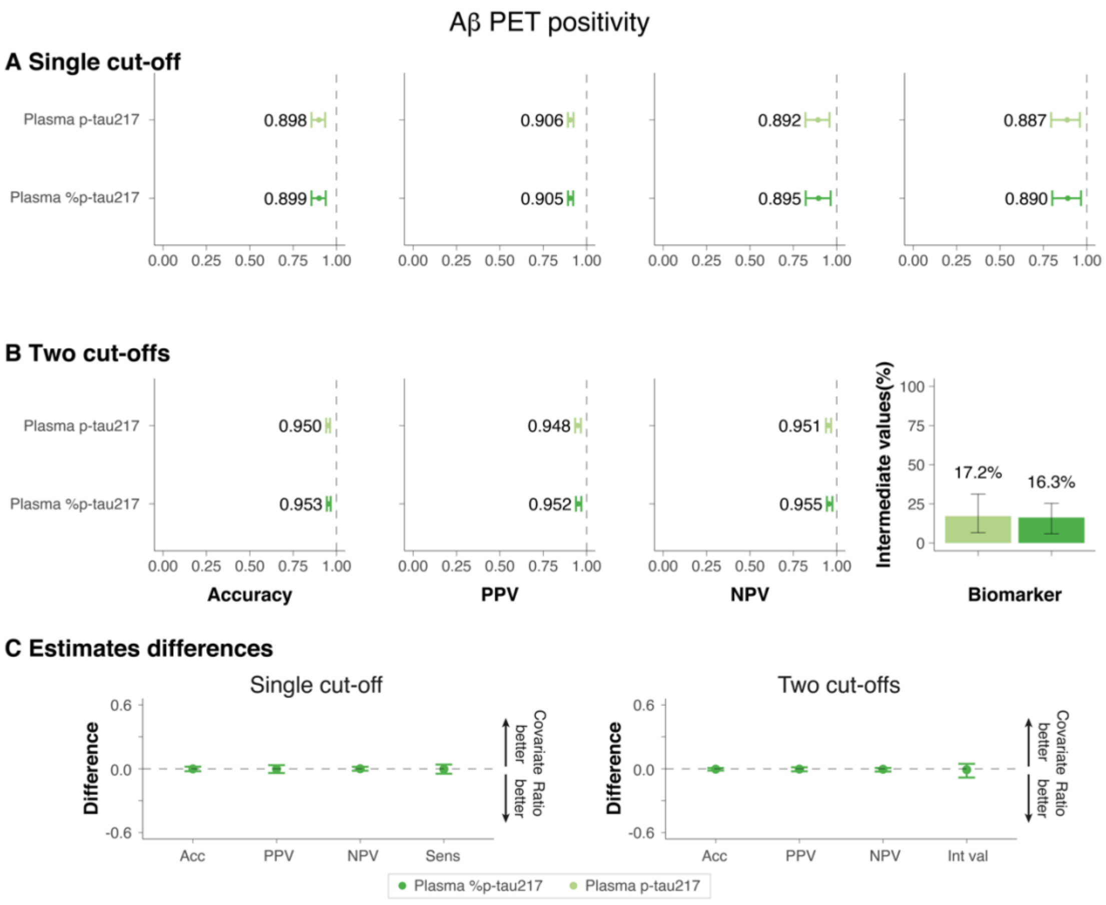

Supplementary Figure 5: Comparison between plasma p-tau217 and %p-tau217 on predicting Aβ PET positivity in cognitively impaired patients of the BioFINDER-2 cohort with in-bag estimates

Prediction of Aβ PET positivity in cognitively impaired participants from the BioFINDER-2 cohort, using a single cut-off (A) and two cut-offs (B) approaches, respectively. In the first approach, the cut-off was calculated maximizing sensitivity fixing specificity at 90%. In the second approach, the lower cut-off was obtained by maximizing specificity with sensitivity fixed at 95%, whereas the upper cut-off was obtained by maximizing sensitivity fixing specificity at 95%. Participants that fall between these two cut-offs were classified in the intermediate group. Dots and error bars represent the actual statistic and 95%CI (from bootstrapped n=1,000 samples with replacement), respectively. The model with plasma p-tau217 as predictor also included the non-phosphorylated tau as covariate. Bootstrapped differences (n=1,000 resamples with replacement stratifying by the output) between the statistics using plasma p-tau217 (reference)

and CSF biomarkers are shown in C for both single and two cut-offs. A horizontal dashed line is plotted at zero representing the lack of difference between plasma and CSF biomarkers. We considered plasma and CSF biomarkers clinically equivalent if the 95%CI of the mean difference included zero and clinically superior if it did not include zero and favored plasma (>0). Differences in number of participants in the intermediate group have been scaled to a maximum of one to be comparable to the other differences. Dots and error bars represent the mean and 95%CI estimate from a bootstrapped sample. Vertical dashed lines represent the maximal statistical value possible (1). For the intermediate values plots, coloured bars represent the actual percentage and error bar the 95%CI. Aβ PET positivity was assessed as Centiloids≥37.

Abbreviations: Aβ, amyloid-β; CI, confidence interval; CSF, cerebrospinal fluid; NPV, negative predictive value; PPV, positive predictive value.

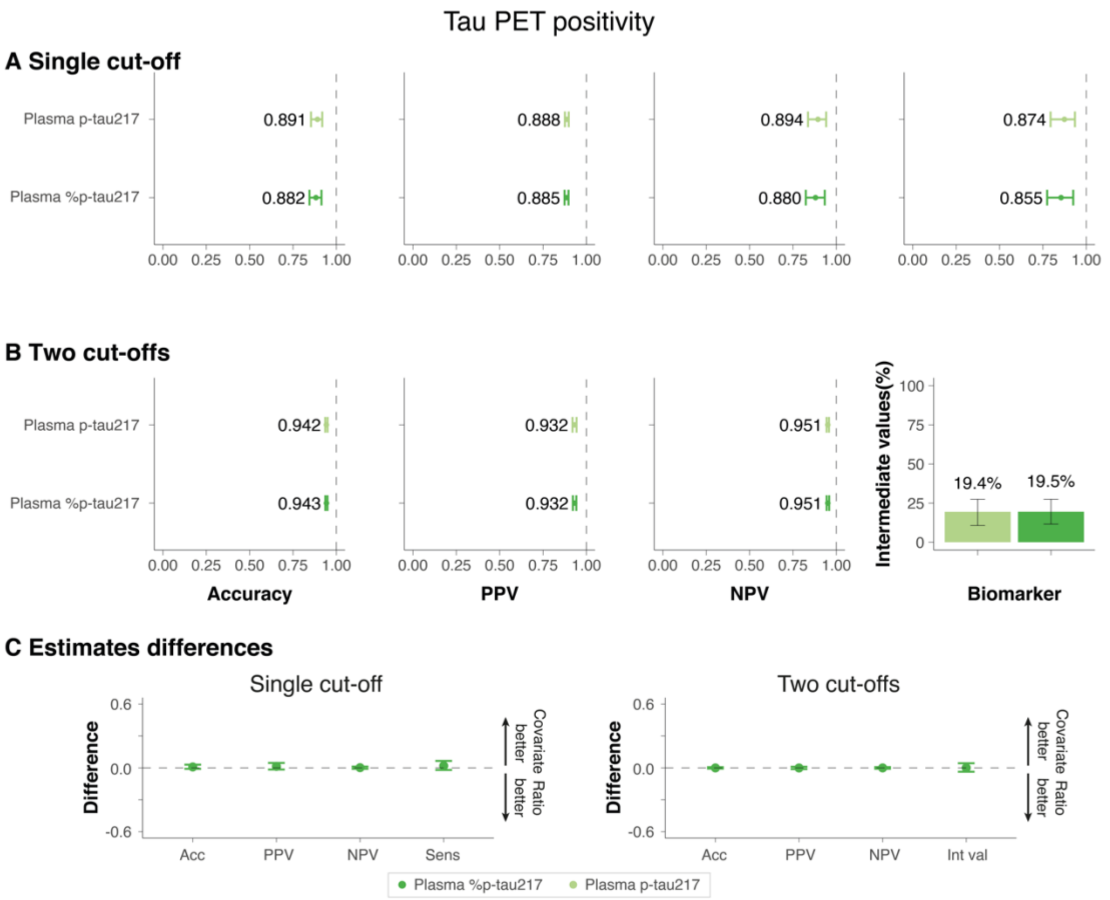

Supplementary Figure 6: Comparison between plasma p-tau217 and %p-tau217 on predicting tau PET positivity in cognitively impaired patients of the BioFINDER-2 cohort with in-bag estimates

Prediction of tau PET positivity in cognitively impaired participants from the BioFINDER-2 cohort, using a single cut-off (A) and two cut-offs (B) approaches, respectively. In the first approach, the cut-off was calculated maximizing sensitivity fixing specificity at 90%. In the second approach, the lower cut-off was obtained by maximizing specificity with sensitivity fixed at 95%, whereas the upper cut-off was obtained by maximizing sensitivity fixing specificity at 95%. Participants that fall between these two cut-offs were classified in the intermediate group. Dots and error bars represent the actual statistic and 95%CI (from bootstrapped n=1,000 samples with replacement), respectively. The model with plasma p-tau217 as predictor also included the non-phosphorylated tau as covariate. Bootstrapped differences (n=1,000 resamples with replacement stratifying by the output) between the statistics using plasma p-tau217 (reference) and CSF biomarkers are shown in C for both single and two cut-offs. A horizontal dashed line is plotted at zero representing the lack of difference between plasma

and CSF biomarkers. We considered plasma and CSF biomarkers clinically equivalent if the 95%CI of the mean difference included zero and clinically superior if it did not include zero and favored plasma (>0). Differences in number of participants in the intermediate group have been scaled to a maximum of one to be comparable to the other differences. Dots and error bars represent the mean and 95%CI estimate from a bootstrapped sample. Vertical dashed lines represent the maximal statistical value possible (1). For the intermediate values plots, coloured bars represent the actual percentage and error bar the 95%CI. Tau PET positivity was assessed using an in-house previously validated cut-off (SUVR>1.32).

Abbreviations: Aβ, amyloid-β; CI, confidence interval; CSF, cerebrospinal fluid; NPV, negative predictive value; PPV, positive predictive value.

	Accuracy		PPV		NPV		Sensitivity	
	**Mean**	**Difference**	**Mean**	**Difference**	**Mean**	**Difference**	**Mean**	**Difference**
**Aβ PET positive**								
**BioFINDER-2**								
Plasma p-tau217	0.90 [0.86, 0.93]	Ref.	0.91 [0.89, 0.92]	Ref.	0.89 [0.82, 0.96]	Ref.	0.89 [0.79, 0.96]	Ref.
Plasma %p-tau217	0.90 [0.86, 0.94]	0.00 [-0.02, 0.02]	0.91 [0.89, 0.92]	0.00 [-0.02, 0.02]	0.90 [0.82, 0.97]	0.00 [-0.04, 0.03]	0.89 [0.80, 0.97]	0.00 [-0.05, 0.04]
**Tau PET positive**								
**BioFINDER-2**								
Plasma p-tau217	0.89 [0.85, 0.92]	Ref.	0.89 [0.88, 0.90]	Ref.	0.89 [0.84, 0.94]	Ref.	0.87 [0.79, 0.93]	Ref.
Plasma %p-tau217	0.88 [0.84, 0.91]	0.01 [-0.01, 0.03]	0.89 [0.87, 0.90]	0.00 [-0.01, 0.01]	0.88 [0.82, 0.93]	0.01 [-0.02, 0.05]	0.85 [0.77, 0.92]	0.02 [-0.02, 0.07]

Supplementary Table 9: Comparison between plasma p-tau217 and %p-tau217 on predicting Aβ and tau PET positivity in cognitively impaired patients using a single cut-off approach with in-bag estimates

Comparison of accuracy, PPV, NPV and sensitivity among fluid biomarkers on predicting Aβ and tau PET positivity in cognitively impaired patients. The cut-off of fluid biomarkers was derived by maximizing sensitivity fixing specificity at 90% against each imaging outcome. The model with plasma p-tau217 as predictor also included the non-phosphorylated tau as covariate. Differences between the statistics using plasma p-tau217 (reference) and CSF biomarkers are shown together with the mean values. We considered plasma and CSF biomarkers clinically equivalent if the 95%CI of the mean difference included zero and clinically superior if it did not include zero and favored plasma (>0). Aβ PET positivity was assessed as Centiloids≥37. Tau PET positivity was assessed using in-house previously validated cut-offs (SUVR>1.32 for both cohorts).

Abbreviations: Aβ, amyloid-β; CI, confidence interval; CSF, cerebrospinal fluid; NPV, negative predictive value; PPV, positive predictive value.

	Accuracy		PPV		NPV		Number of Intermediate participants	
	Mean	Difference	Mean	Difference	Mean	Difference	Mean	Difference*
**Amyloid PET positive**								
**BioFINDER-2**								
Plasma p-tau217	0.95[0.94,0.96]	Ref.	0.95[0.93,0.97]	Ref.	0.95[0.94,0.97]	Ref.	17.2 [6.6,31.3]	Ref.
Plasma %p-tau217	0.95[0.94,0.97]	0.00 [-0.02, 0.01]	0.95[0.94,0.97]	0.00 [-0.02, 0.01]	0.96[0.94,0.98]	0.00 [-0.03, 0.01]	16.3 [5.9,25.3]	-0.01 [-0.08, 0.05]
**Tau PET positive**								
**BioFINDER-2**								
Plasma p-tau217	0.94[0.94,0.95]	Ref.	0.93[0.92,0.94]	Ref.	0.95[0.94,0.96]	Ref.	19.4 [10.7,27.5]	Ref.
Plasma %p-tau217	0.94[0.94,0.95]	0.00 [-0.01, 0.01]	0.93[0.92,0.94]	0.00 [-0.01, 0.01]	0.95[0.94,0.96]	0.00 [-0.01, 0.01]	19.5 [11.6,27.5]	0.00 [-0.04, 0.04]

Supplementary Table 10: Comparison between plasma p-tau217 and %p-tau217 on predicting Aβ and tau PET positivity in cognitively impaired patients using a two cut-offs approach with in-bag estimates

Comparison of accuracy, PPV, NPV and number participants categorised in the intermediate group among fluid biomarkers on predicting Aβ and tau PET positivity in cognitively impaired patients. The lower cut-off was obtained by maximizing specificity with sensitivity fixed at 95%, whereas the upper cut-off was obtained by maximizing sensitivity fixing specificity at 95%. Participants that fall between these two cut-offs were classified in the intermediate group. The model with plasma p-tau217 as predictor also included

the non-phosphorylated tau as covariate. Differences between the statistics using plasma %p-tau217 (reference) and CSF biomarkers are shown together with the mean values. *Differences in number of participants in the intermediate group has been scaled to a maximum of one to be comparable to the other differences. We considered plasma and CSF biomarkers clinically equivalent if the 95%CI of the mean difference included zero and clinically superior if it did not include zero and favored plasma (>0). Aβ PET positivity was assessed as Centiloids≥37. Tau PET positivity was assessed using in-house previously validated cut-offs (SUVR>1.32 for both cohorts).

Abbreviations: Aβ, amyloid-β; CI, confidence interval; CSF, cerebrospinal fluid; NPV, negative predictive value; PPV, positive predictive value.

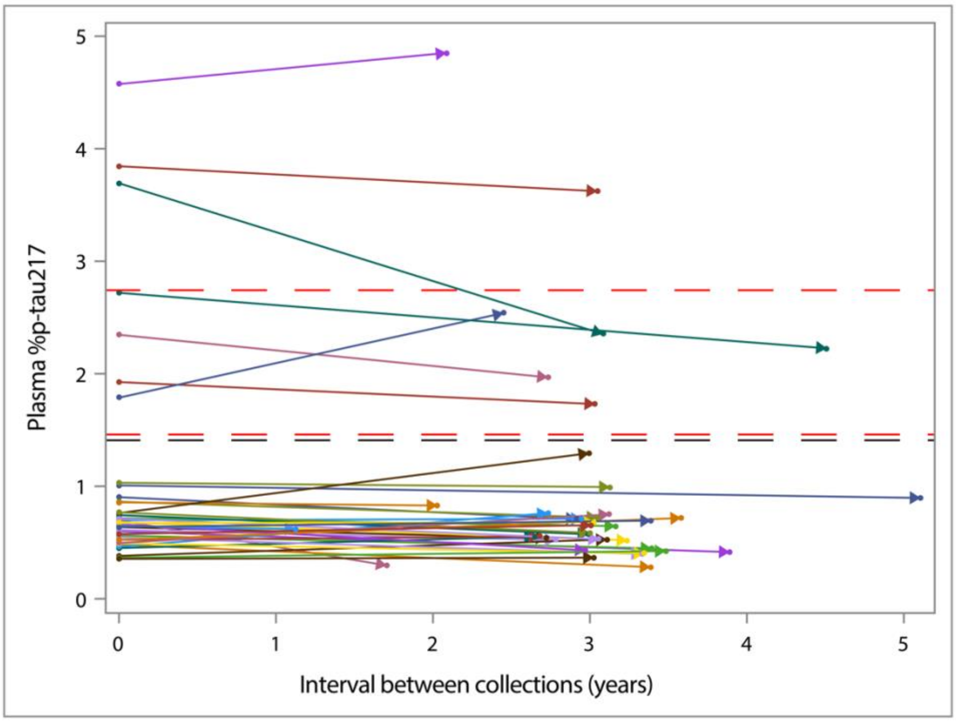

Supplementary Figure 7: Longitudinal trajectories of plasma %p-tau217 in Knight ADRC participants

Individual longitudinal trajectories of plasma %p-tau217 in 40 participants of the Knight ADRC cohort. All participants except one (2.5%) remained in the same Aβ category using the cut-off obtained in our main analyses. Horizontal dashed black line represents the threshold derived from the single cutoff approach, and red lines represent the lower and upper thresholds from the two cutoffs approach.

Supplementary Methods

Plasma %p-tau217 analysis by IP-MS

Plasma samples were thaw on Thermomixer (ThermoFisher Scientific) at 21°C under 1000rpm rotation for 10min then centrifuged at 4°C 30,000rpm for 30min. 900µl of plasma supernatant free of precipitate and lipid layer was transferred into an Axygen (Corning) 1.7ml polypropylene tube then transferred to 96-DeepWell Plates (Waters, Milford, MA). 20µl of 0.125 ng/µl 15N-Tau labelled internal standard (Promise-Proteomics, Grenoble, France) in 0.1% SigMatrix Serum Diluent (Sigma Aldrich) then 50µl of a mixture containing 10% NP-40, 10X Complete Protease Inhibitor cocktail (Roche) and 50mM Guanidine were added before mixing for 2hrs at room temperature. 30µl of a mixture of Dynabeads Epoxy- 270 (Invitrogen) respectively coupled with Tau1 (tau epitope 192-199, Kanaan Lab, Michigan State University, MI), HJ8.5 and HJ8.7 (epitopes respectively 27- 35 and 118-122, Holtzman Lab, Washington University School of Medicine, MO) antibodies was added to the samples. Plate was shaken at 4°C 1000 rpm for 20hrs. Beads containing immunoprecipitated plasma tau were washed 3 times with 1ml solution of 25mM tetraethylammonium bicarbonate (TEABC). Tau protein was eluted from the beads using 200µl 0.1% trifluoro acetic acid (TFA) solution. The eluate was transferred to Oasis HLB µelution plate (Waters, Milford, MA) previously conditioned with 200µl methanol then 2x200µl 0.1% TFA, and desalted with 2x200µl 0.1%TFA. Tau was eluted to a 96-dwell plate using 27.5% acetonitrile (ACN) 0.1%TFA and eluate was dried on Speedvac (Labconco). After adding 5µl of a mixture of AQUA peptides internal standards (ThermoFisher Scientific) containing 0.5fmol phosphorylated and 5fmol non phosphorylated in 0.005% SigMatrix, samples were digested for 16 hours at 37°C with 400ng of trypsin (Promega). Digests were loaded on Oasis HLB µelution plate conditioned with 200µl methanol, 200µl 60%ACN 0.1% formic acid (FA) then 2x200µl 0.1%FA, desalted with 2x200µl 0.1% FA and eluted with 200µl 60%ACN 0.1%FA. Eluate was dried using Speedvac resuspended in 30µl 2%ACN 0.1%FA, centrifuged at 30,000 rpm then 25µl of the solution was transferred to MS vial before drying on Speedvac. Samples were stored at 4°C before resuspension in 12.5µl 0.1%FA prior to analysis. Tau digests were analyzed by liquid chromatography and tandem high resolution mass

spectrometry (LC-MS/HRMS) on a nanoAcquity UPLC system (Waters, Mildford, Massachusetts) coupled to an Orbitrap Tribrid Eclipse MS (Thermo Scientific, San Jose, California) operating in Parallel Reaction Monitoring mode. 4.5µl of sample extract was loaded on a 75µm x 100µm nanoEase HSS C18 T3 100A, 1.8µm nanoLC column (Waters, Milford, MA) at a flow rate of 0.4µl/min of 0.5%ACN 0.1%FA for 7min. Plasma samples were run in batches of approximately 80 samples together with plasma quality controls from plasma pools aliquots at low and high level of p-tau217 to assess intra- and inter-plate measurement reproducibility. MS/HRMS transitions were extracted using Skyline version 22.2.2.278 (MacCoss lab, University of Washington). LC-MS data were aggregated to calculate %p-tau217 using the ratio between LC-MS/HRMS signals from TPSLPpTPPTR 212-221 phosphorylated peptide at T217 and unmodified peptide TPSLPTPPTR. Phosphorylated to non-phosphorylated peptide endogenous ratio was normalized using the ratio measured on the MS/HRMS transitions of the corresponding AQUA phosphorylated/non-phosphorylated peptide internal standards. All data extraction steps were performed by operators blinded to any clinical or biomarker information regarding the samples.

Comparison of plasma %p-tau217 measurements in Knight ADRC and BioFINDER-2

Plasma samples were processed and analyzed by LC-MS into batches of approximately 80 samples. BioFINDER-2 and Knight ADRC cohorts were analyzed during two separated sessions of 21 and 8 batches respectively. To estimate plasma p-tau measure variation into and between batches and across analyses sessions, samples aliquots from two plasma pools with low and high values of %p-tau217 were included in each batch as quality controls. Two biological replicates of each low and high plasma p-tau quality controls were analyzed for each batch.

For plasma %p-tau217 cut-offs cross-validation between the two cohorts, the BioFINDER-2 dataset values were adjusted to fit the Knight ADRC dataset using two points calibration method. The calibration used the comparison of the means measured on low and high p-tau quality controls from the Knight ADRC and

BioFINDER-2 sessions to evaluate measures differences between the sessions then correct the BioFINDER-2 using Knight ADRC measures as reference.

UCSF cohort

The University of California San Francisco (UCSF) Alzheimer's Disease Research Center cohort (n=251) was used to derive the CSF cut-offs for the Elecsys platform in an independent cohort from BioFINDER-2, for a sensitivity analysis. Details from this cohort can be found in [1]. In summary, Aβ PET was acquired either with [^{11}C]PIB or with [^{18}F]AV45. CSF was obtained following ADNI protocols[2], and collected and measured as explained in [3] in three different centers. Cut-offs in this cohort were derived using the same approach that in BioFINDER-2 and Knight ADRC using visual read Aβ PET positivity as outcome, Only impaired participants were included following the same approach as in the main analyses. Demographic and other characteristics of the cohort are shown in Supplementary Table 11.

UCSF			
	**All (n=251)**	**VR negative (n=140)**	**VR positive (n=111)**
Age, years	63.2 (8.73)	63.1 (8.92)	63.4 (8.51)
Women, n (%)	123 (49.0%)	63 (45.0%)	60 (54.1%)
APOE-ε4 carriers, n (%)[a]	82 (32.7%)	32 (22.9%)	50 (45.0%)
Years of education[b]	16.8 (3.20)	16.7 (3.35)	16.9 (3.02)
MMSE[c]	23.5 (5.58)	24.5 (5.46)	22.4 (5.54)
Aβ PET, Centiloids[d]	44.1 (52.2)	1.14 (12.3)	95.1 (31.3)
Aβ PET positive, n (%)[d]	102 (40.6%)	2 (1.4%)	100 (90.1%)

Supplementary Table 11: UCSF participants' characteristics

All measures represent mean (SD) unless otherwise stated. Percentages are calculated from the sample available for each variable, Visual read (VR) was used to assess Aβ PET positivity. Additionally, Aβ PET positivity by quantification was defined as Centiloids≥37.

[a] 23 participants missing, [b] 7 participants missing, [c] 12 participants missing, [d] 12 participants missing.

References

1. Leuzy A, Mattsson-Carlgren N, Cullen NC, et al. Robustness of CSF Aβ42/40 and Aβ42/P-tau181 measured using fully automated immunoassays to detect AD-related outcomes. Alzheimer's & Dementia 2023;19(7):2994–3004.
2. Shaw LM, Vanderstichele H, Knapik-Czajka M, et al. Cerebrospinal fluid biomarker signature in Alzheimer's disease neuroimaging initiative subjects. Ann Neurol 2009;65(4):403–13.
3. Bittner T, Zetterberg H, Teunissen CE, et al. Technical performance of a novel, fully automated electrochemiluminescence immunoassay for the quantitation of β-amyloid (1-42) in human cerebrospinal fluid. Alzheimers Dement 2016;12(5):517–26.
