# Supplementary material for: Highly accurate blood test for Alzheimer’s disease is similar or superior to clinical cerebrospinal fluid tests
Source: Nat Med. 2024 Feb 21;30(4):1085–95. doi: 10.1038/s41591-024-02869-z (PMC11031399; doi:10.1038/s41591-024-02869-z)
Supplement: Supplementary file 2 — Reporting Summary [file 41591_2024_2869_MOESM2_ESM.pdf]

nature portfolio

Corresponding author(s): Randall J Bateman
Oskar Hansson

Last updated by author(s): Jan 30, 2024

Reporting Summary

Nature Portfolio wishes to improve the reproducibility of the work that we publish. This form provides structure for consistency and transparency in reporting. For further information on Nature Portfolio policies, see our Editorial Policies and the Editorial Policy Checklist.

Statistics

For all statistical analyses, confirm that the following items are present in the figure legend, table legend, main text, or Methods section.

n/a	Confirmed	
☐	☒	The exact sample size (n) for each experimental group/condition, given as a discrete number and unit of measurement
☐	☒	A statement on whether measurements were taken from distinct samples or whether the same sample was measured repeatedly
☐	☒	The statistical test(s) used AND whether they are one- or two-sided<br>*Only common tests should be described solely by name; describe more complex techniques in the Methods section.*
☐	☒	A description of all covariates tested
☐	☒	A description of any assumptions or corrections, such as tests of normality and adjustment for multiple comparisons
☐	☒	A full description of the statistical parameters including central tendency (e.g. means) or other basic estimates (e.g. regression coefficient) AND variation (e.g. standard deviation) or associated estimates of uncertainty (e.g. confidence intervals)
☒	☐	For null hypothesis testing, the test statistic (e.g. F, t, r) with confidence intervals, effect sizes, degrees of freedom and P value noted<br>*Give P values as exact values whenever suitable.*
☒	☐	For Bayesian analysis, information on the choice of priors and Markov chain Monte Carlo settings
☒	☐	For hierarchical and complex designs, identification of the appropriate level for tests and full reporting of outcomes
☒	☐	Estimates of effect sizes (e.g. Cohen's d, Pearson's r), indicating how they were calculated

Our web collection on statistics for biologists contains articles on many of the points above.

Software and code

Policy information about availability of computer code

Data collection	NanoLC-MS/MS experiments were performed using nanoAcquity UPLC system (Waters, Mildford, MA) coupled to Orbitrap Eclipse mass spectrometer (Thermo Scientific, San Jose, CA). MS/MS transitions peaks were extracted and quantified using Skyline-daily v23.1.1 (MacCoss Lab, Department of Genome Sciences, University of Washington, Seattle, WA).
Data analysis	R (v4.1.0) was used for statistical analyses as described in the manuscript. The following packages were used: pROC, cutpointr and boot.

For manuscripts utilizing custom algorithms or software that are central to the research but not yet described in published literature, software must be made available to editors and reviewers. We strongly encourage code deposition in a community repository (e.g. GitHub). See the Nature Portfolio guidelines for submitting code & software for further information.

Data

Policy information about availability of data

All manuscripts must include a data availability statement. This statement should provide the following information, where applicable:

- Accession codes, unique identifiers, or web links for publicly available datasets
- A description of any restrictions on data availability
- For clinical datasets or third party data, please ensure that the statement adheres to our policy

For BioFINDER-2 data, anonymized data will be shared by request from a qualified academic investigator for the sole purpose of replicating procedures and results presented in the article and as long as data transfer is in agreement with EU legislation on the general data protection regulation and decisions by the Ethical

Review Board of Sweden and Region Skåne, which should be regulated in a material transfer agreement. For Knight ADRC data are available to qualified investigators who have a proposal approbed by an institutional committee (https://knightadrc.wustl.edu/Research/ResourceRequest.htm) that meets monthly. the study must be approved by an institutional review board to ensure ethical reseearch practives and investigators must agree to the terms and conditions of the data use agreement, which includes not distributing the data without permission.

Human research participants

Policy information about studies involving human research participants and Sex and Gender in Research.

Reporting on sex and gender	Sex is reported in Table 1.
Population characteristics	Sample characteristics are shown in Table 1
Recruitment	n BioFINDER-2, the sample consisted of patients that had been referred to participting memory clinics (mostly from primary care) and most cognitively unimpaired participants were recruited from the general population in the south of Sweden.Knight ADRC cohort consisted of community-dwelling volunteers enrolled in studies of memory and aging at Washington University in St. Louis. All Knight ADRC participants underwent a comprehensive clinical assessment that included a detailed interview of a collateral source, a neurological examination of the participant, the Clinical Dementia Rating (CDR®) and the MMSE. Individuals with a CDR of 0.5 or greater were considered to have a dementia syndrome and the probable aetiology of the dementia syndrome was formulated by clinicians based on clinical features in accordance with standard criteria and methods.
Ethics oversight	All participants gave written informed consent and ethical approval was granted by the Regional Ethical Committee in Lund, Sweden and the Washington University Human Research Protection Office, respectively.

Note that full information on the approval of the study protocol must also be provided in the manuscript.

Field-specific reporting

Please select the one below that is the best fit for your research. If you are not sure, read the appropriate sections before making your selection.

☒ Life sciences ☐ Behavioural & social sciences ☐ Ecological, evolutionary & environmental sciences

For a reference copy of the document with all sections, see nature.com/documents/nr-reporting-summary-flat.pdf

Life sciences study design

All studies must disclose on these points even when the disclosure is negative.

Sample size	The study included two prospective studies (BioFINDER-2 [n=1,422] and Knight ADRC [n=337]) with large sample sizes. All participants with available plasma %p-tau217 and CSF Ab42/40 and CSF p-tau181/Ab42 measures at baseline were analyzed in this study.
Data exclusions	There was no data exclusion in this study.
Replication	We replicated key findings in two large independent cohorts (BioFINDER-2 and Knight ADRC) with significant differences in demographics and outcome measures.
Randomization	There was no randomization in this study. We did not perform any adjustment for covariates due to the comparison of biomarkers within the same sample.
Blinding	CSF and plasma measurments were performed blinded to the clinical data.

Reporting for specific materials, systems and methods

We require information from authors about some types of materials, experimental systems and methods used in many studies. Here, indicate whether each material, system or method listed is relevant to your study. If you are not sure if a list item applies to your research, read the appropriate section before selecting a response.

Materials & experimental systems

n/a	Involved in the study
☐	☒ Antibodies
☒	☐ Eukaryotic cell lines
☒	☐ Palaeontology and archaeology
☒	☐ Animals and other organisms
☐	☒ Clinical data
☒	☐ Dual use research of concern

Methods

n/a	Involved in the study
☒	☐ ChIP-seq
☒	☐ Flow cytometry
☒	☐ MRI-based neuroimaging

Antibodies

Antibodies used

Tau1 (generated by Drs Nicholas Kanaan) and HJ series (HJ8.5, HJ8.7, HJ32.11 and HJ34.8) antibodies (generated by Dr. David Holtzman) were used. Detailed information of the immunoassays in the manuscript has been published previously (and is referred to in the manuscript).

Validation

Tau1, HJ8.5 and HJ8.7 were validated in the following studies:
-Barthélemy NR, et al. Site-specific cerebrospinal fluid tau hyperphosphorylation in response to Alzheimer's disease brain patholgy: Not all tau phospho-sites are hyperphosphorylated. Journal of Alzheimer's disease, 2022, 85(1): 415-29.
-Sato, et al. Tau kinetic in neurons and the human central nervous sytem. Neuron 2018. 98(4): 861-4.
HJ32.11 and HJ34.8 were newly generated antibodies and we confirmed that immunoprecipitation procedures using there antibodies worked well by the two replicate cohorts analyses.

Clinical data

Policy information about clinical studies
All manuscripts should comply with the ICMJE guidelines for publication of clinical research and a completed CONSORT checklist must be included with all submissions.

Clinical trial registration

NCT03174938 (BioFINDER-2)

Study protocol

For BioFINDER-2: www.biofinder.se.

Data collection

BioFINDER-2 data are collected at the memory clinics of Skåne University Hospital and Ängelholm's hospital in Sweden between April 2017 and June 2022. Participants in Knight ADRC cohort were community-dwelling volunteers enrolled in studies of memory and aging between February 2013 and March 2020.

Outcomes

The primary outcome is amyloid status assessed by PET. Secondary outcomes include tau status assessed by PET and Alzheimer's disease diagnosis.
